# Structural and Functional Studies of a Phosphatidic Acid-Binding Antifungal Plant Defensin MtDef4: Identification of an RGFRRR Motif Governing Fungal Cell Entry

**DOI:** 10.1371/journal.pone.0082485

**Published:** 2013-12-04

**Authors:** Uma Shankar Sagaram, Kaoutar El-Mounadi, Garry W. Buchko, Howard R. Berg, Jagdeep Kaur, Raghu S. Pandurangi, Thomas J. Smith, Dilip M. Shah

**Affiliations:** 1 Donald Danforth Plant Science Center, Saint Louis, Missouri, United States of America; 2 Biological Sciences Division, Pacific Northwest National Laboratory, Richland, Washington, United States of America; Bernhard Nocht Institute for Tropical Medicine, Germany

## Abstract

MtDef4 is a 47-amino acid cysteine-rich evolutionary conserved defensin from a model legume *Medicago truncatula*. It is an apoplast-localized plant defense protein that inhibits the growth of the ascomycetous fungal pathogen *Fusarium graminearum in vitro* at micromolar concentrations. Little is known about the mechanisms by which MtDef4 mediates its antifungal activity. In this study, we show that MtDef4 rapidly permeabilizes fungal plasma membrane and is internalized by the fungal cells where it accumulates in the cytoplasm. Furthermore, analysis of the structure of MtDef4 reveals the presence of a positively charged γ-core motif composed of β_2_ and β_3_ strands connected by a positively charged RGFRRR loop. Replacement of the RGFRRR sequence with AAAARR or RGFRAA abolishes the ability of MtDef4 to enter fungal cells, suggesting that the RGFRRR loop is a translocation signal required for the internalization of the protein. MtDef4 binds to phosphatidic acid (PA), a precursor for the biosynthesis of membrane phospholipids and a signaling lipid known to recruit cytosolic proteins to membranes. Amino acid substitutions in the RGFRRR sequence which abolish the ability of MtDef4 to enter fungal cells also impair its ability to bind PA. These findings suggest that MtDef4 is a novel antifungal plant defensin capable of entering into fungal cells and affecting intracellular targets and that these processes are mediated by the highly conserved cationic RGFRRR loop via its interaction with PA.

## Introduction

Defensins are small cysteine-rich proteins present in all plants and constitute an ancient and diverse set of natural antimicrobial proteins. These 45- to 54-residue proteins usually contain four intramolecular disulfide bonds and adopt a similar cysteine-stabilized α/β (CSα/β) motif in which one α-helix is stabilized through disulfide bridging to a three-strand antiparallel β-sheet [1,2]. Because plant defensins lack a distinct hydrophobic core, the protein fold is stabilized primarily by the disulfide bonds. Although structural features of plant defensins are highly conserved, a comparison of their primary amino acid sequences reveals a rich diversity of variants [2,3]. This sequence diversity is responsible for the functional diversity observed in defensins [4,5].

Several plant defensins exhibit antifungal activity *in vitro* at low micromolar concentrations, [2-4]. These defensins also display differing antifungal properties. Morphogenic defensins inhibit hyphal elongation with a concomitant increase in hyphal branching, whereas nonmorphogenic defensins reduce hyphal elongation without causing significant morphological changes [6,7]. Because of their potent antifungal activity, defensins have been widely exploited in agrobiotechnological applications to generate disease resistant crops. Indeed, transgenic plants overexpressing defensins exhibit resistance to a range of fungal and oomycete pathogens [8]. In order to fully harness the potential of these proteins for bioengineering crops with robust resistance to fungal and oomycete pathogens, it is essential to understand their structure-activity relationships and modes of antifungal action. 

Nearly two decades of studies have revealed that plant defensins interact with fungal-specific cell wall and plasma membrane components and inhibit fungal growth from the extracellular or intracellular side of fungal cells [9,10]. For example, *Raphanus sativus* AFP2 (RsAFP2) and *Dahlia merckii* AMP1 (DmAMP1) bind with high affinity to distinct sphingolipids present in the plasma membrane or cell wall of their target fungi. Such interactions are a prerequisite for antifungal activity [11-13]. RsAFP2 interacts with glucosylceramide (GlcCer) present in the cell wall causing accumulation of apoptosis-inducing ceramides [14], whereas DmAMP1 binds specifically to mannosyl diinositolphosphoryl ceramide present in the plasma membrane [15]. *Medicago sativa* defensin 1 (MsDef1) is also thought to interact with GlcCer since a *Fusarium gramine*arum mutant ∆Fg*gcs1* lacking GlcCer exhibits strong resistance to this defensin [16]. In some instances, a defensin must be internalized by fungal cells after cell wall binding to cause cell death [17,18]. For Nicotiana alata defensin 1 (NaD1), this process is mediated by the premeabilization of fungal hyphae using a cell wall-dependent process [19]. Once inside fungal cells, these defensins likely target intracellular processes critical for fungal growth. For example, *Pisum sativum* defensin 1 (Psd1) is active against the model filamentous fungus *Neurospora crassa* and once inside the cells of this fungus it interacts with a nuclear cyclin-like protein involved in cell-cycle control and co-localizes in the nuclei [17]. The precise mechanisms of the antifungal action of defensins capable of entry into fungal cells are still not fully understood and the sequence motifs mediating fungal cell entry of these defensins remain to be identified.

To date, all known antifungal plant defensins containing disulfide bonds possess a highly conserved γ-core motif (GXCX_3–9_C, where X is any amino acid) consisting of β2 and β3 strands with an interposed loop that carries a net positive charge and participates in one to four disulfide bonds [20]. Structure-activity studies indicate that the major determinants of antifungal activity reside in the γ-core motif [21,22].

MtDef4 is an evolutionarily conserved defensin from *M. truncatula* and its homologs are found in all plants examined to date [22,23]. MtDef4 carries a net positive charge of + 6. It inhibits the growth of several filamentous fungi including *F. graminearum* at micromolar concentrations. Since it does not induce hyperbranching of fungal hyphae, it is thought to be a nonmorphogenic defensin [24]. Homology-based structure of MtDef4 predicts a γ-core motif GRCRGFRRRC composed of the β2 and β3 strands connected by the RGFRRR loop. When the γ-core motif of a related defensin MsDef1 is replaced by the γ-core motif of MtDef4, the resulting chimeric defensin MsDef1-γ4 exhibits antifungal properties similar to MtDef4 [22]. In addition, chemically synthesized γ-core peptide of MtDef4 also inhibits the growth of *F. graminearum in vitro*, further substantiating the importance of this motif in the antifungal action of this defensin [22]. 

In this study, we have shown that MtDef4 is internalized by fungal cells. The atomic structure of MtDef4 has also been determined. The structure-activity studies of MtDef4 have established the RGFRRR loop of the γ-core motif as a translocation signal mediating entry of this defensin into fungal cells. The RGFRRR loop interacts with the phospholipid phosphatidic acid (PA) and this interaction is likely required for the entry of MtDef4 into fungal cells and/or its intracellular toxicity.

## Results

### MtDef4 enters the cytoplasm of F. *graminearum*

**Figure 1:**
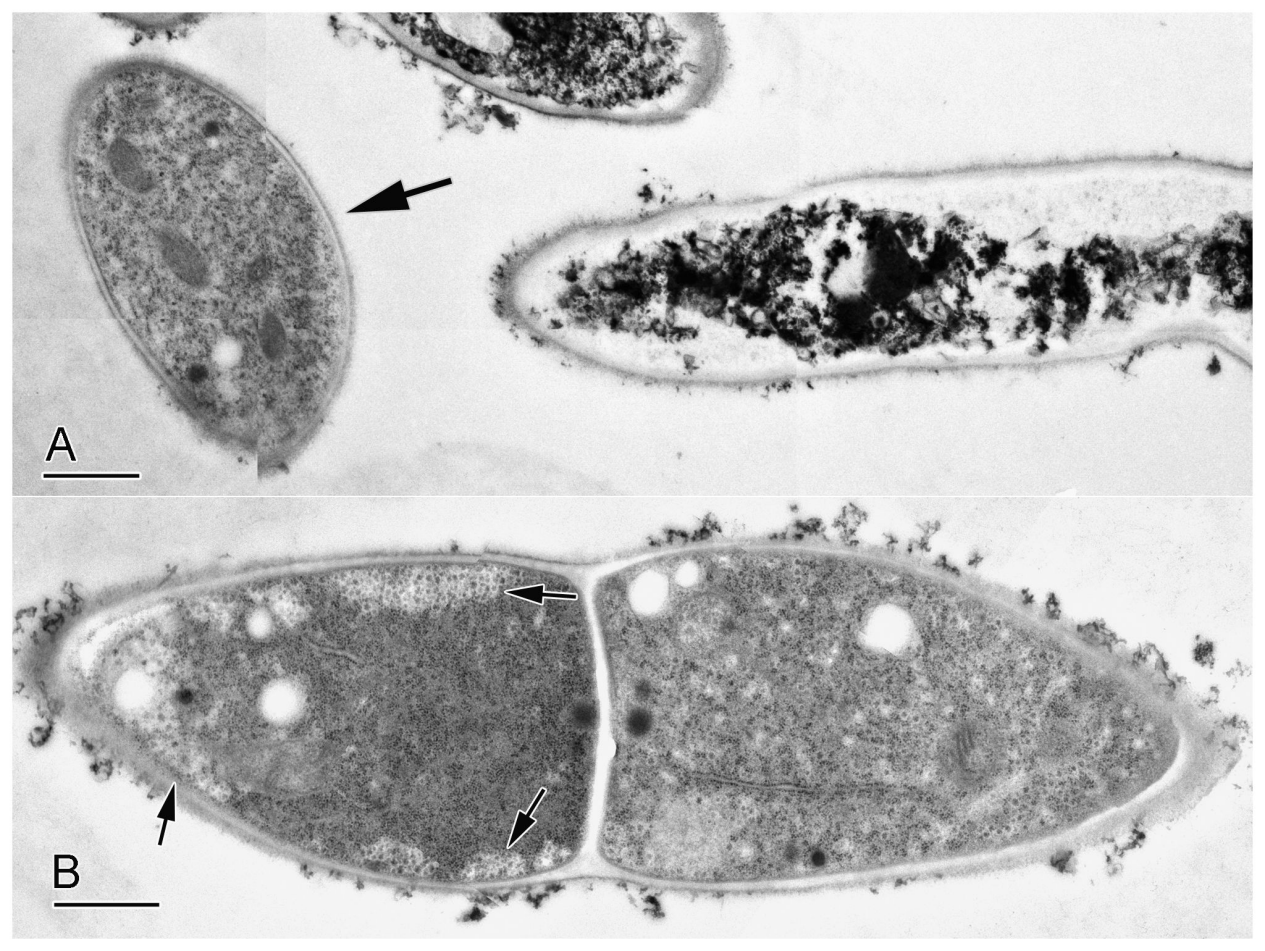
Ultrastructure of MtDef4-treated cells of *Fusarium* *graminearum*. Cells were treated with 3µM MtDef4 for three hrs. Scale bars = 1 µm. (A) Cells were a mixed population with dead and live cells (arrow) both present. (B) Two adjacent cells in a hypha with the left cell in early stages of MtDef4-mediatd cell degradation. The cortical cytoplasm is separating from the cell wall (arrows) and is more electron dense than the healthier cell on the right.

MtDef4 permeabilizes the plasma membrane of *F. graminearum* in a concentration dependent manner [22]. We therefore sought to determine if MtDef4 is internalized by *F. graminearum* cells using immunogold labeling and transmission electron microscopy (TEM) of freeze substituted material. Figure 1 shows that *F. graminearum* hyphal cells treated with 3 µM MtDef4 were heterogeneous both in ultrastructure and labeling by anti-MtDef4 antibody. Their ultrastructure ranged from cells similar to the control cells (Figure 1A, arrow) to those whose structure was disrupted (other cells in Figure 1A) with degraded, aggregated, and electron dense cytoplasm. Of the two cells in the hypha shown in Figure 1B, the cell on the right was healthy but the cell on the left was beginning to show signs of deterioration, including increased electron density and segregation of cytoplasm from the cell periphery (arrows). This was unlikely to be an artifact of preparation because the cells were ultra-rapidly frozen (i.e., fixed in ~10 msec), dehydrated and embedded while frozen.

**Figure 2:**
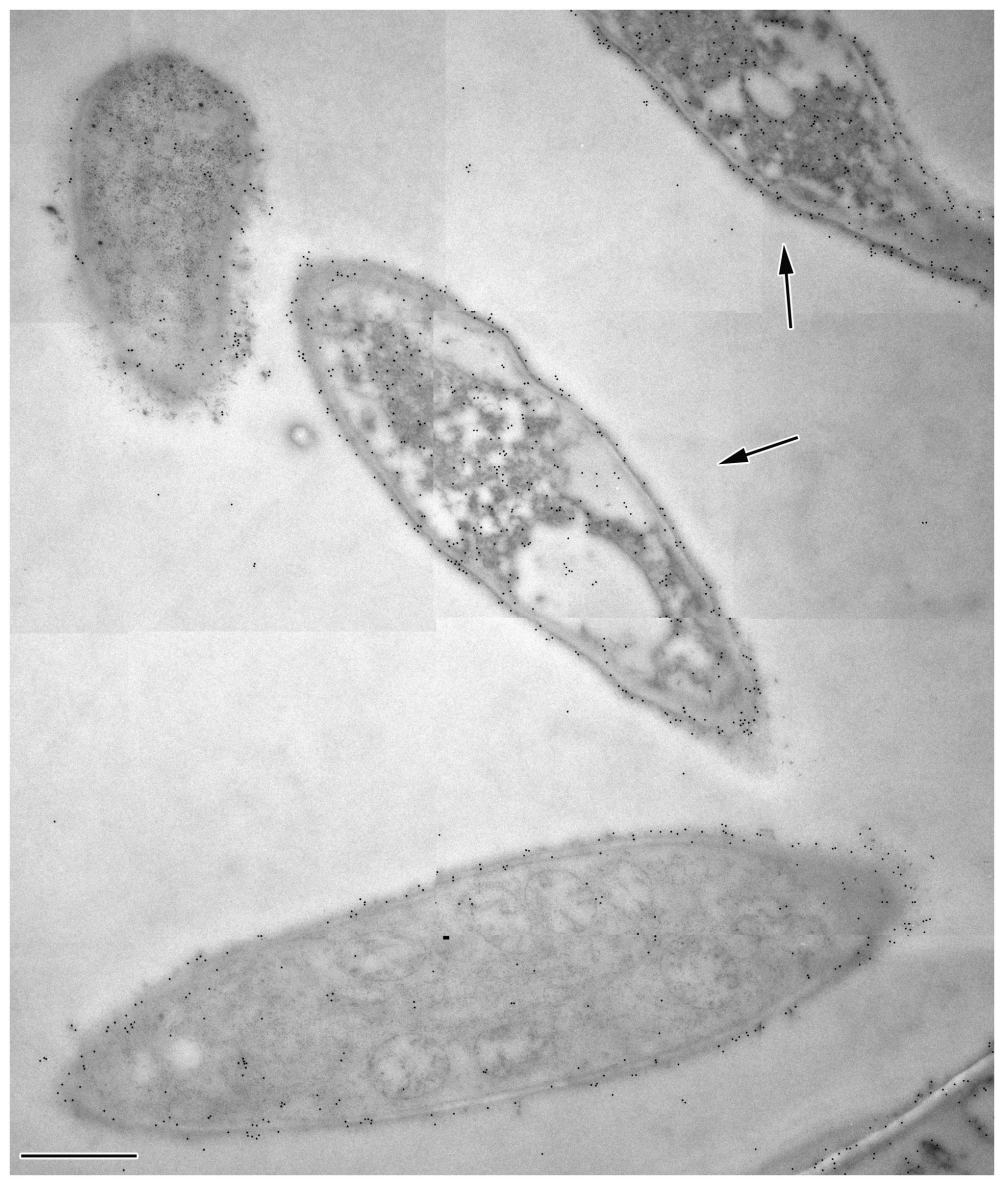
Immunogold detection of MtDef4 in treated cells (3 µm, 3 hours) of *F*. ***graminearum***. Scale bar = 1 µm. Section was not post-stained. Of the four cells shown in this section, the two dead cells (arrows) have significantly higher cytoplasmic labeling than the two living cells.

**Figure 3:**
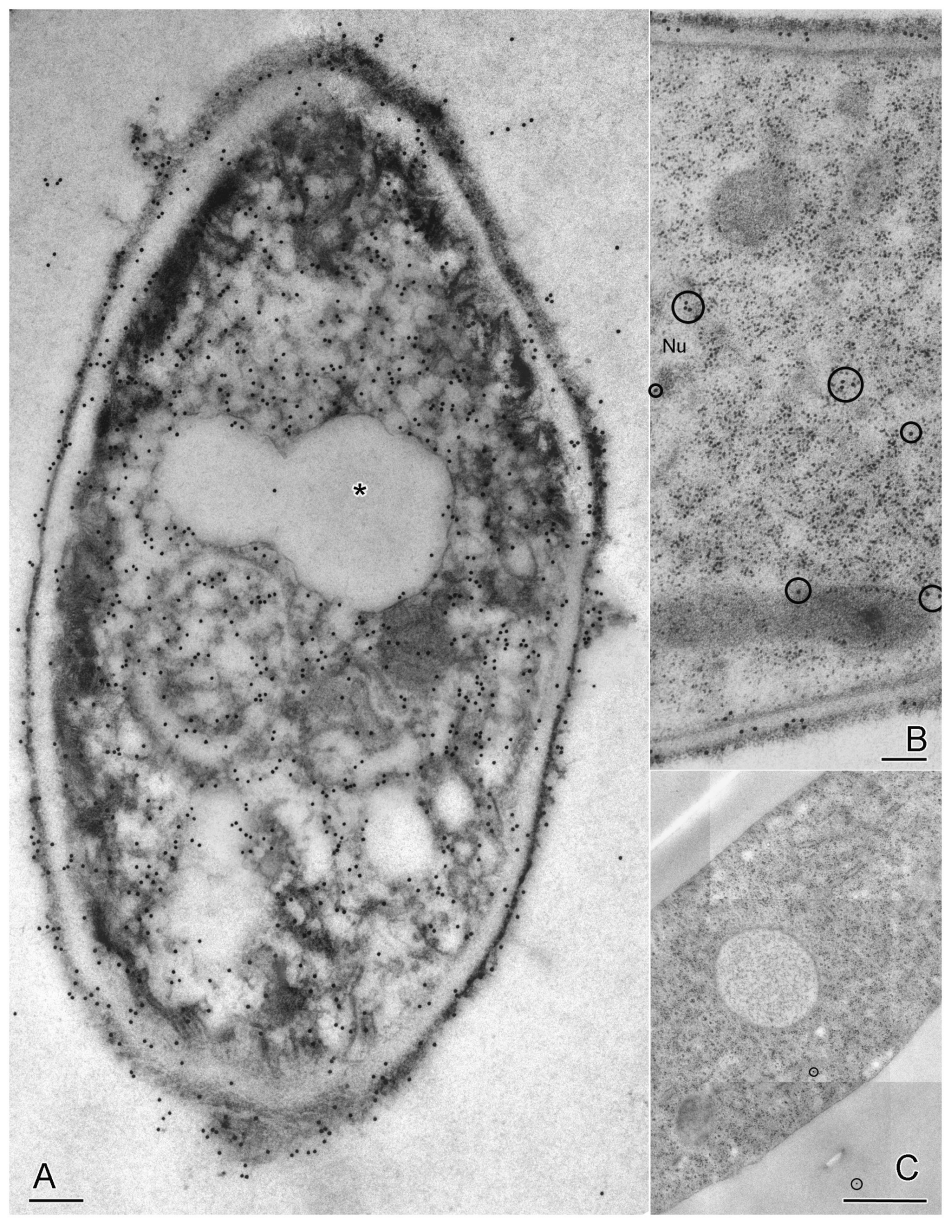
Immunogold label distribution in MtDef4-treated cells. Scale bars = 250 nm (A, B), 1 µm (C). A, B are of post-stained sections, C is not. (A) In dead cells cytoplasmic label is associated with electron dense aggregated cytoplasm of undetermined cellular structure. Vacuole (asterisk) does not label. In these cells cytoplasmic label density is 95 particles per µm^2^ while that of cell wall is 312 particles per µm^2^. (B) Cells not yet killed by MtDef4 show a small amount of MtDef4 in the cytoplasm (circled gold particles) but much more in the cell wall. (C) Control cells treated with water alone are not labeled (two gold particles circled are background). Label density over the cytoplasm was the same as on resin alone, 0.04 particles per µm^2^ (+ /-) 0.04).

**Figure 4:**
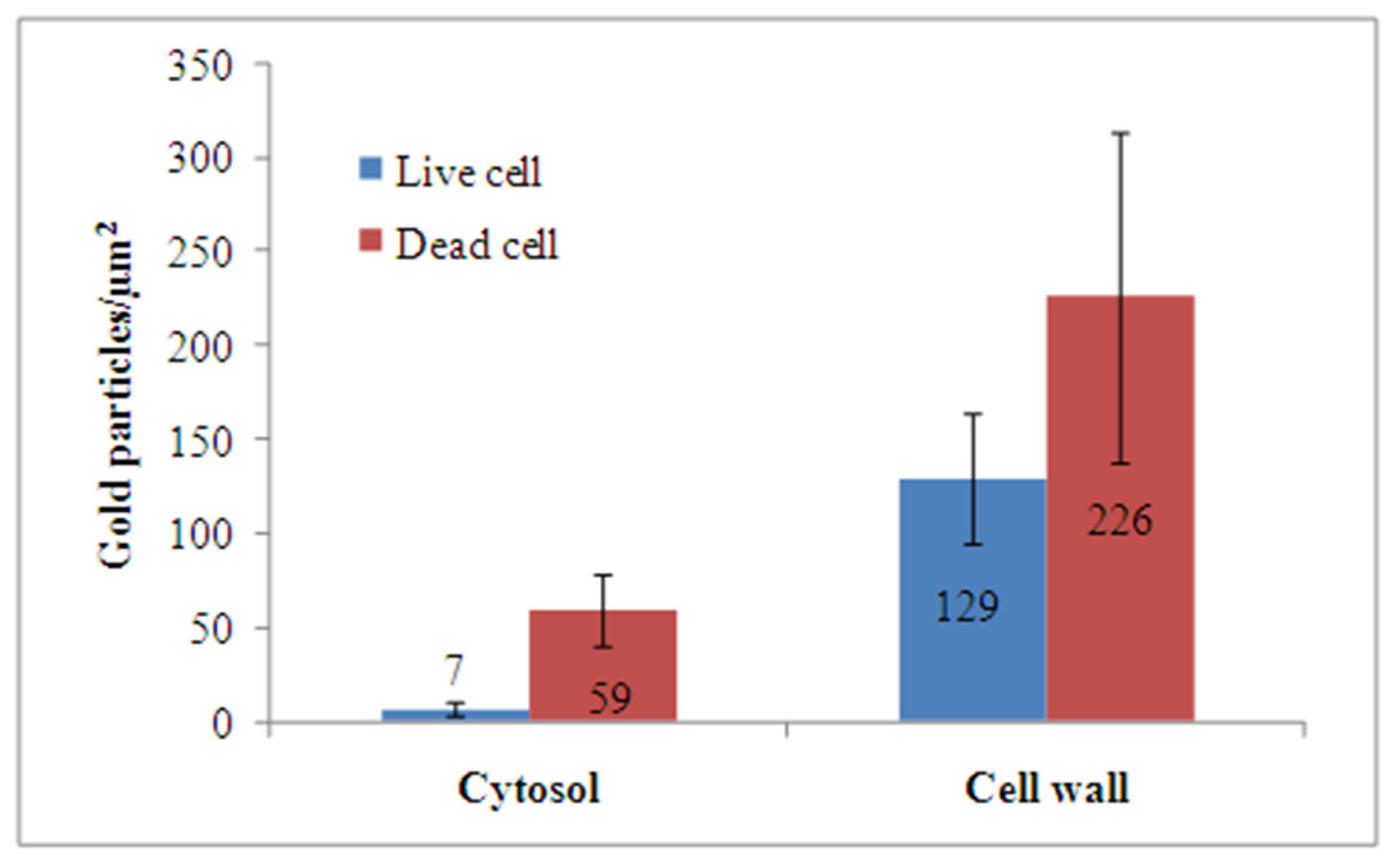
Number of gold particles found in the cytoplasm and cell walls of live and dead cells of *F*. ***graminearum***. The error bars represent the standard errors of six replicates.

When sections of these samples were immunogold labeled with anti-MtDef4 antibody, there was an inverse correlation between quality of ultrastructure and density of anti-MtDef4 immunogold label in the cytoplasm. Thus, the dead cells in Figure 2 (arrows) had a higher labeling density. This would be expected if MtDef4 that entered the cell caused this cytoplasmic degradation. It is noteworthy that cells showing healthy ultrastructure contained MtDef4 in their cell wall and a few gold particles in their cytoplasm, suggesting they were in the early stages of invasion by MtDef4. The electron dense cytoplasm in dead cells was aggregated (Figures 2, 3A), leaving voids labeled rarely with gold. Vacuoles in these cells were also not labeled (Figure 3A, asterisk). Label in the electron dense aggregates in dead cells was not associated with any obvious cell structure (Figure 3A). The cytosol in the cell in Figure 3A with the densest labeling contained 759 gold particles at a density of 95 particles per µm^2^. Living cells had fewer cytoplasmic gold particles (Figure 3B, circled). Control cells, treated with water only, showed ultrastructure that was expected in healthy *F. graminearum* cells and they uniformly lacked labeling with anti-MtDef4 antibody (Figure 3C, the two circled gold particles were non-specific labeling). The number of gold particles per µm^2^ was measured in 25 living vs. degraded cells and on cell walls vs. cytoplasm (Figure 4). Cytoplasm labeling in living cells averaged 7 particles per µm^2^ vs. 59 for degraded cells, and for cell wall labeling there was a similar trend i.e., 129 in living vs. 226 in degraded cells. The high standard deviation for living cell cytoplasmic labeling reflects varying degrees of ingress of MtDef4 while in degraded cells it reflects a large variation in cytoplasmic aggregation. As noted above, anti-MtDef4 antibody labeled aggregates but rarely labeled voids surrounding aggregates that were included in the area measurement. The background label in cell-free areas of the same section was 1.3+ /-1.6 particles per µm^2^ and label density in cells treated with water only was not above background of 0.04+ /-0.04 particles per µm^2^. Since the ratio of cell wall label to cytoplasm label decreased almost five fold in living cells compared to degraded cells (Figure 4), it seems that MtDef4 first binds to the cell wall and then enters the cytoplasm. Since the volume of the cytoplasmic compartment is much larger than that of the cell wall, the ratio of cytoplasmic gold particles in degraded cells to those in living cells of 8.4 is perhaps relevant.

### Structural and modeling analysis of MtDef4 reveals a surface-exposed patch of positively charged amino acids derived from the γ-core motif

**Figure 5:**
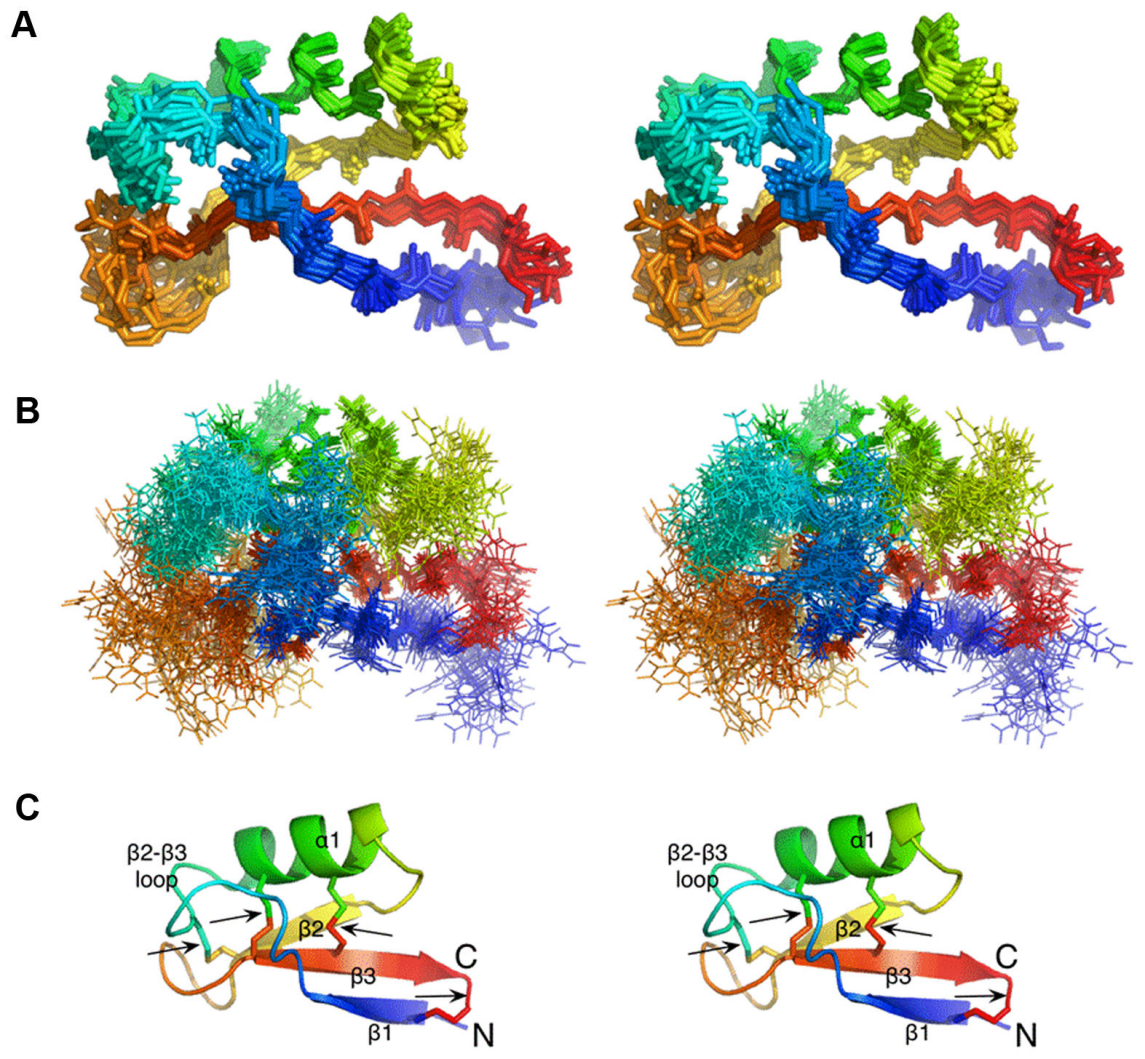
NMR structure of MtDef4. A. Backbone superposition of the top 20 refined MtDef4 structures. Shown here are only the backbone atoms of the various structures colored from blue to red as the chains extend from the amino-terminus to carboxy-terminus. B. Superposition of the top 20 structures showing all of the atoms in the models. The orientation and coloration is the same as in A. C. Ribbon diagram of the MtDef4 mean structure in the same color and orientation as above. The disulfide bonds are noted by arrows and the amino- and carboxy-termini are labeled.

**Figure 6:**
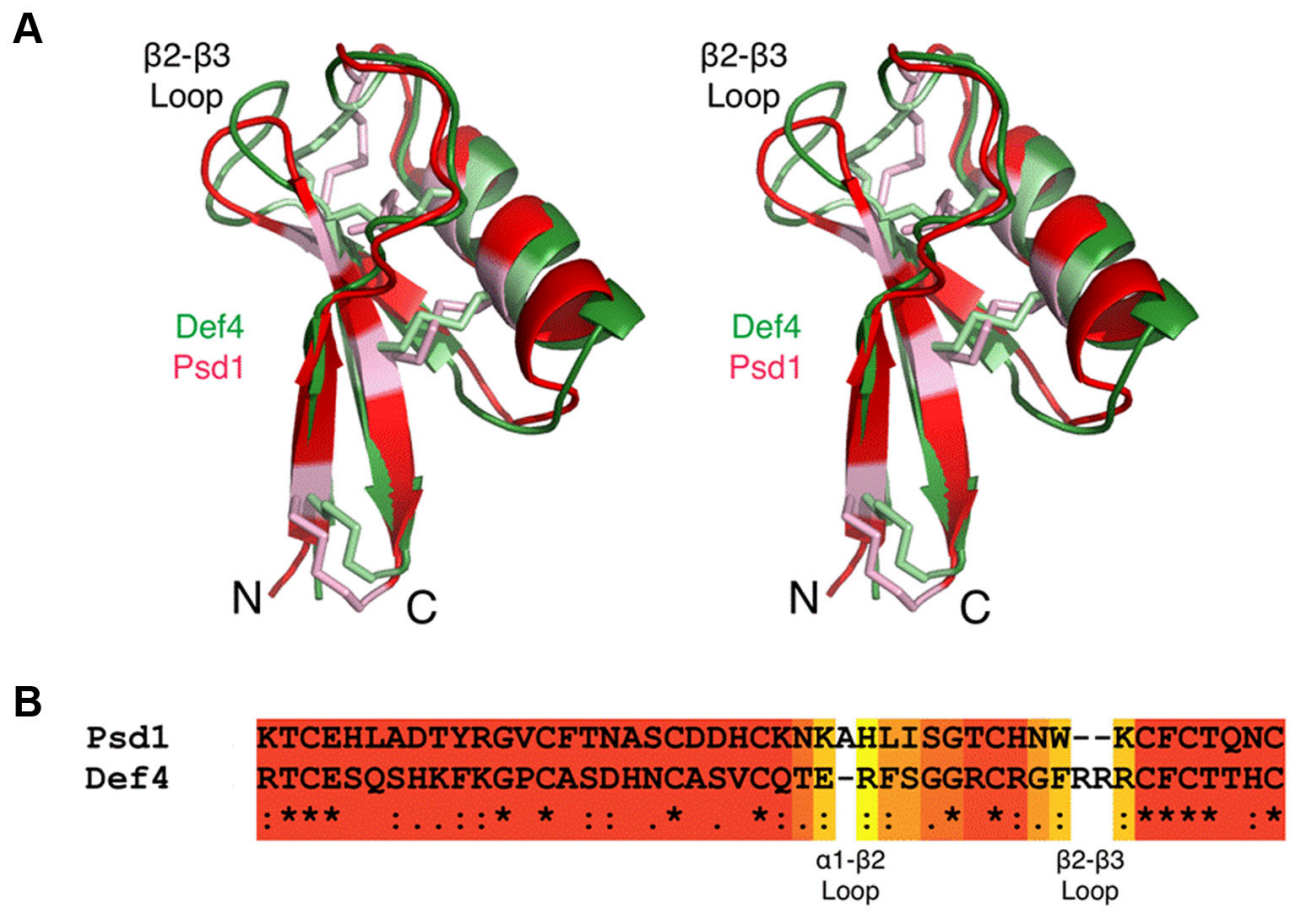
Structural homology between MtDef4 and other plant defensins. A. Shown here is the structural alignment of MtDef4 and the plant defensin Psd1 from *Pisum* *sativum* (PDB code 1JKZ) in green and red, respectively. The cysteine residues involved in the four disulfide bonds are highlighted in lighter hues. B. Sequence alignments based on the 3D alignments using the program EXPRESSO [27]. The coloring of the alignments ranges from blue to red as the error in the alignment goes from high to low. Note that the β2-β3 loop in MtDef4 is longer by two residues and is far more basic than that in Psd1. However, the exposed hydrophobic F37 residue in MtDef4 is highly similar to W38 residue in Psd1.

To determine the structural basis for the biochemical function of the γ-core motif, an NMR-based solution structure was determined for MtDef4 and its structure compared to other homologous defensins. As shown in Figure 5, MtDef4 adopts a highly compact structure composed of a three-strand (β1=X2-X6; β2=X31-X35; β3=X40-X46) anti-parallel β-sheet with a single α-helix (α1=X17-X27) between β1 and β2. The protein is held together by four intramolecular disulfide bonds C3-C47, C14-C34, C20-C41 and C24-C43. When compared to the plant defensin, Psd1, which is known to be internalized by fungal cells [25,26], MtDef4 has an identical core structure with regard to folding and locations of the highly conserved disulfide bonds (Figure 6A). As with all plant defensins whose tertiary structures have been determined (data not shown), the majority of the structural and sequence differences lie in the loop connecting α1 and β2 and the loop connecting β2 and β3. As previously noted [25], the amino acid sequence homology is far weaker than the structural homology amongst members of this family of proteins. Therefore, the sequences of Psd1 and MtDef4 were aligned incorporating structural homology using the program EXPRESSO [27]. As shown in Figure 6B, the residues highlighted in red represent those that align very well between the two structures. As expected, the α1-β2 and β2-β3 loops are the only areas with significant differences. Interestingly, the β2-β3 loop in Psd1 is shorter by two residues than the corresponding loop in MtDef4 and this loop is shorter in MtDef4 than in other plant defensins such as RsAFP2. These results suggest that the major difference between plant defensins lie in the small loop between β2 and β3, the γ-core motif. 

**Figure 7:**
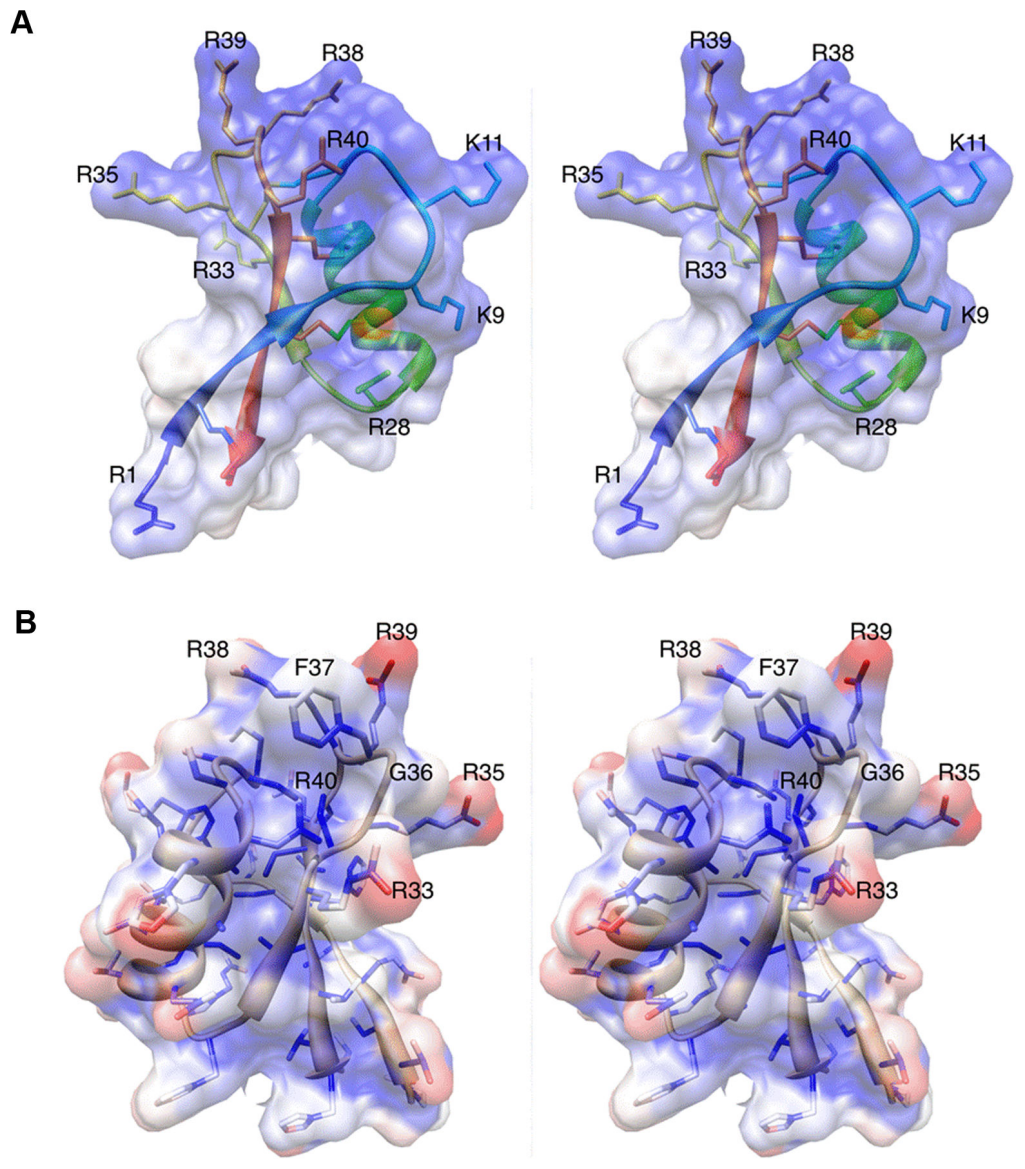
Surface characteristics of MtDef4. A. In this diagram, the surface potential (calculated by DelPhi) is mapped onto the molecular surface of MtDef4 as a semi-transparent surface. The structure of MtDef4 is represented by a ribbon diagram that is colored blue to red as the chain extends from the amino-terminus to carboxy terminus. The disulfide bonds and the side chains of the arginine and lysine residues in the structure are also shown. Note that the overall surface is strongly basic. B. Surface accessibility of the atoms in the MtDef4 structure. The semi-transparent molecular surface is colored blue to red for the least to most accessible atoms. Note that the very hydrophobic F37 on the β2-β3 loop, which is highly conserved among defensin proteins, is significantly exposed. The residues in this β2-β3 loop region are labeled.

MtDef4 has an unusual distribution of surface residues that are likely important in facilitating its fungal cell entry and antifungal action. As shown in Figure 7, MtDef4 has a large number of arginine residues that are clustered around the β2-β3 loop. Using the program DELPHI [28,29], the electrostatic potential was calculated for MtDef4 using only whole charges and then mapped onto the molecular surface using the program Chimera [30]. As shown in Figure 7A, the surface of MtDef4 is entirely positively charged with residues of the RGFRRR loop derived from its γ-core motif. This is in contrast to a number of other related defensins and toxins that show more of a distribution of acidic and basic residues on the surface [25]. 

The third residue of the RGFRRR loop is a conserved hydrophobic amino acid present in the MtDef4 homologs of higher plants [23]. It seemed likely that this residue, F37, should be buried away from the solvent exposed surface because it is surrounded by positively charged amino acids. Instead, the aromatic ring of F37 is oriented away from the protein’s core. To further examine this, the degree of solvent accessibility for each atom was calculated with the program AREAIMOL [31,32] within the CCP4i [33] suite of programs. The degree of exposure of each atom was then mapped onto the molecular surface using the program Chimera [30]. As shown in Figure 7B, over half of the aromatic side chain of F37 is exposed to the solvent. As discussed below, mutagenesis analysis suggests that the nature and location of this residue is important for the antifungal activity of MtDef4.

### RGFRRR loop mediates antifungal activity of MtDef4

**Figure 8:**
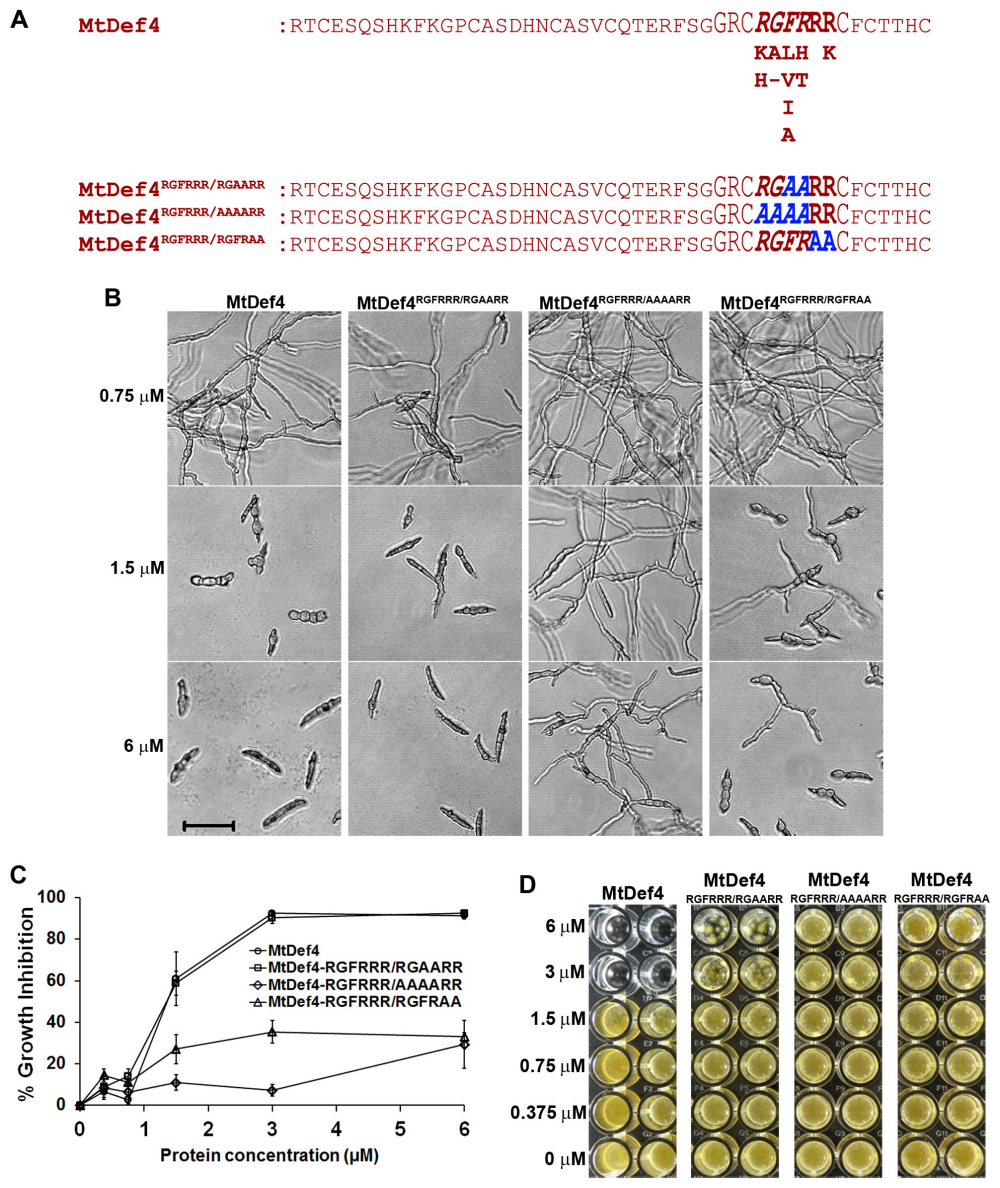
The RGFRRR loop present in the γ-core motif strongly regulates the antifungal activity of MtDef4. A. Sequence of MtDef4 and its variants. The γ-core motif is indicated in larger font. RGFRRR sequence of MtDef4 is indicated in bold and the conserved amino acids are listed underneath with highly conserved amino acids on the top followed by less conserved ones. RGFR sequence which closely resembles the RXLR motif of the fungal and oomycete effectors is italicized. B. Images showing the inhibition of *F*. *graminearum* PH-1 conidial germination and hyphal growth at different concentrations of MtDef4 or its variants. Images were taken after 16 hours of incubation of conidia with defensins. Bar = 50 μm. C. Quantitative assessment of the *in* *vitro* antifungal activity of MtDef4 or its variants at 4 days after incubation of PH-1 conidia with defensins. Values are means of thee replications. Error bars indicate standard deviations. D. Images showing the growth of PH-1 strain after 6 days in the presence of MtDef4 or its variants.

Previously, the γ-core motif of MtDef4 was identified as an important structural determinant of its antifungal activity [22]. We showed that replacement of the γ-core motif of MsDef1 with that of MtDef4 substantially increased the antifungal activity of MsDef1. Also, studies with synthetic peptides derived from the carboxy-terminus of MtDef4 revealed that the cationic and hydrophobic amino acids present in the γ-core motif were important for antifungal activity [22]. Based on the NMR structure of MtDef4, we hypothesized that the RGFRRR loop present within the γ-core motif plays a critical role in the fungal cell entry and/or the intracellular toxicity of this defensin. The RGFR sequence of this loop closely resembles the well-studied RxLR motif present in the oomycete effectors translocated into plant cells [34-36]. In fact, we have noticed that a couple of MtDef4 homologs of cassava contain the RxLR motif (Figure S1). We therefore mutagenized RGFR motif to AAAA by generating the MtDef4^RGFRRR/AAAARR^ variant and tested its fungal cell entry and antifungal activity. A second variant, MtDef4^RGFRRR/RGAARR^, was tested since a previously tested synthetic peptide RGAARR had significantly reduced antifungal activity when compared with the RGFRRR peptide [22]. A third variant, MtDef4^RGFRRR/RGFRAA^, was generated to determine the role C-terminal RR residues play in the fungal cell entry and antifungal activity of MtDef4. MtDef4 and three variants (Figure 8A; Table 1) were each expressed in *Pichia pastoris* and purified as described in Experimental procedures. Mass spectrometric analysis demonstrated that all three MtDef4 variants had expected mass and formed four disulfide bonds (data not shown). Furthermore, they also had very similar circular dichroism (CD) spectra indicating that the amino acid substitutions did not impact the overall protein fold and secondary structure composition (Figure S2). The ability of the variants to inhibit conidial germination and hyphal growth was compared to that of MtDef4 [37]. *F. graminearum* conidia were incubated in the presence of different concentrations of MtDef4 or its variants and the conidial germination and hyphal growth were monitored and measured at 16, 24, 36, 48, 60 and 72 hrs. These assays revealed that MtDef4^RGFRRR/RGAARR^ had slightly reduced activity and the other 2 variants MtDef4^RGFRRR/AAAARR^ and MtDef4^RGFRRR/RGFRAA^ had significantly reduced activity, when compared to MtDef4 (Figure 8B, C and D). MtDef4^RGFRRR/RGAARR^ variant uniformly inhibited the conidial germination similar to MtDef4 for 24 hr (Figure 8B). At 36 hr after incubation, however, a small proportion of conidia (< 5%) were able to germinate and produce hyphal extensions at 3 µM of this variant, whereas conidial germination was inhibited 100% by MtDef4 at this concentration (Figure S3). MtDef4^RGFRRR/AAAARR^ was the least effective in inhibiting conidial germination. In presence of 6 µM of this variant, almost all conidia were able to germinate and produce hyphal tips resulting in a meager 29 ± 11% growth inhibition at 4 days (Figure 8B). The variant, MtDef4^RGFRRR/RGFRAA^, was as potent as MtDef4 in inhibiting conidial germination at a concentration of 1.5 µM and higher (Figure 8B); however, an obvious breakdown in the activity was noticed at 36 hr and later (Figure 8C and D). At 48 hr, this variant had higher antifungal activity than MtDef4^RGFRRR/AAAARR^ but significantly less than MtDef4 and MtDef4^RGFRRR/RGAARR^ (data not shown). At 4 days and later, MtDef4^RGFRRR/RGFRAA^ exhibited higher potency than MtDef4^RGFRRR/AAAARR^ at 1.5 and 3 µM concentrations, but not at 6 µM (Figure 8C). The difference in growth inhibition caused by MtDef4 and MtDef4^RGFRRR/RGAARR^ became obvious only at 4 days (data not shown) and by 6 days prolific growth of mycelium was obvious at 3 µM and 6 µM of MtDef4^RGFRRR/RGAARR^ treatment. The same concentrations of MtDef4 exhibited 100% growth inhibition (Figure 8D). Since alanine substitutions may change physicochemical properties of MtDef4, two additional variants containing more conservative amino acid substitutions, MtDef4^RGFRRR/KMIKRR^ and MtDef4^RGFRRR/KMIKRK^ were generated. However, when expressed in P. pastoris, these variants were found to be unstable and thus full length variants with correct mass could not be recovered. Collectively, these data indicate that the N-terminal RGFR and C-terminal RR residues of the RGFRRR loop are important for the antifungal activity of MtDef4 and that F37 and R38 residues make only small contribution to the antifungal activity of MtDef4.

**Table 1:** List of proteins/peptides used, their amino acid sequences, net charge and growth inhibitory concentrations against *F. graminearum*.

**Name**	**Amino acid sequence**	**^#^Charge**	**^&^IC_100_**
MtDef4	RT**C**ESQSHKFKGP**C**ASDHN**C**ASV**C**QTERFSGGR**C**RGFRRR**C**F**C**TTH**C**	+ 6.1	3
MtDef4^RGFRRR/RGAARR*^	RT**C**ESQSHKFKGP**C**ASDHN**C**ASV**C**QTERFSGGR**C**RG*AA*RR**C**F**C**TTH**C**	+ 5.1	>6
MtDef4^RGFRRR/AAAARR*^	RT**C**ESQSHKFKGP**C**ASDHN**C**ASV**C**QTERFSGGR**C** *AAAA*RR**C**F**C**TTH**C**	+ 4.1	>6
MtDef4^RGFRRR/RGFRAA*^	RT**C**ESQSHKFKGP**C**ASDHN**C**ASV**C**QTERFSGGR**C**RGFR*AA* **C**F**C**TTH**C**	+ 4.1	>6
MsDef1	RT**C**ENLADKYRGP**C**FSG**C**DTH**C**TTKENAVSGR**C**RDDFR**C**W**C**TKR**C**	+ 3.1	>6
MsDef4-γ4	RT**CE**NLADKYRGP**C**FSG**C**DTH**C**TTKENAVSGR**C** *RGFRRR* **C**W**C**TKR**C**	+ 7.1	3-4
GMA4-C	GR**C**RGFRRR**C**F**C**TTH**C**	+ 5.1	< 6
TMR-GMA4-C	TAM-GR**C**RGFRRR**C**F**C**TTH**C**	NA	48
TMR-GMA4-CM	TAM-GR**C***AAAA*RR**C**F**C**TTH**C**	NA	>96

^#^ Net charge of the peptide was estimated (at pH < 7.4; 7.4 = pH of SFM used to conduct assays) using Biochemistry Online- http://vitalonic.narod.ru/biochem/index_en.html

^&^ Amount of protein (µM) required to inhibit *F. graminearum* conidial germination completely in SFM.

^*^ Replaced amino acid(s) arein italics.

Cysteines are inbold.

### RGFRRR loop is required for fungal cell entry of MtDef4

**Figure 9:**
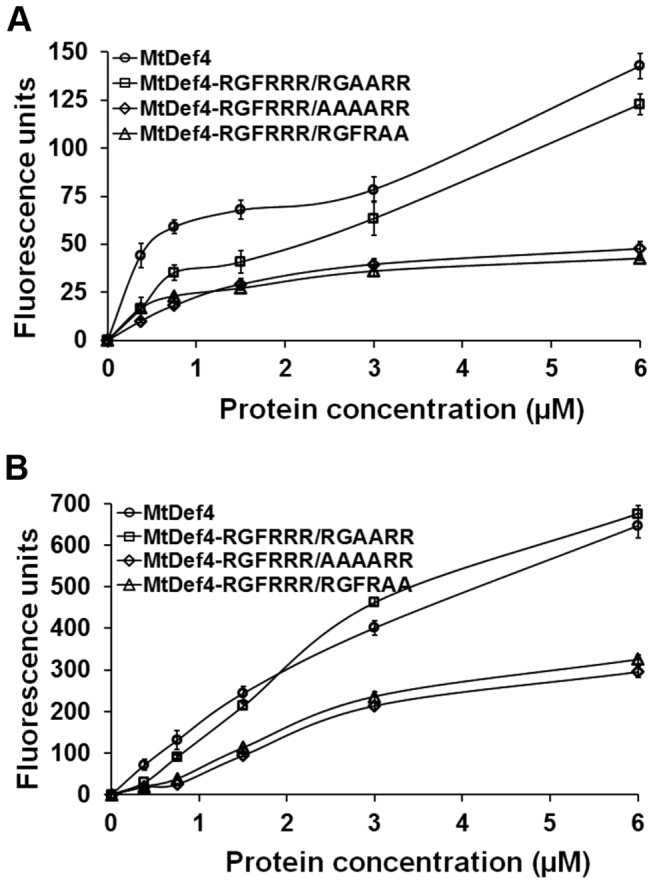
MtDef4 variants, MtDef4^RGFRRR/AAAARR^ and MtDef4^RGFRRR/RGFRAA^, are less efficient in permeabilizing *F*. ***graminearum* membrane compared to MtDef4 or MtDef4^RGFRRR/RGAARR^**. Quantitative measurement of fluorescence emitted by hyphae treated with different concentrations of MtDef4 or its variants plus 0.5 µM of SYTOX Green. Values are means of three replications. Error bars indicate standard deviations. A and B. Fluorescence measurement at 30 min and 8 hr, respectively.

Since MtDef4 is internalized by fungal cells, the question arises as to whether RGFRRR loop also plays a pivotal role in fungal cell entry of MtDef4. In order to answer this question, three MtDef4 variants were first tested for their ability to permeabilize fungal plasma membrane using the SYTOX Green (SG) uptake assay (Sagaram et al., 2011). The fluorescence emitted by SG, taken up by *F. graminearum* hyphae exposed to various concentrations of MtDef4 and its variants was measured at several time points. MtDef4 caused higher permeation of fungal plasma membrane than all three variants at time points of less than 1 hr (Figure 9A). However, at time points of more than 1 hr, the permeation caused by MtDef4^RGFRRR/RGAARR^ was similar to that caused by MtDef4 at all concentrations tested (data not shown). In contrast, MtDef4^RGFRRR/AAAARR^ and MtDef4^RGFRRR/RGFRAA^ permeabilized fungal plasma membrane significantly less than MtDef4 (Figure 9B). No fluorescence was emitted by hyphae in the absence of a defensin and SG. These data indicate that RGFRRR loop plays a role in MtDef4’s ability to permeabilize fungal plasma membrane. 

### RGFRRR loop is required for fungal cell entry of MtDef4

**Figure 10:**
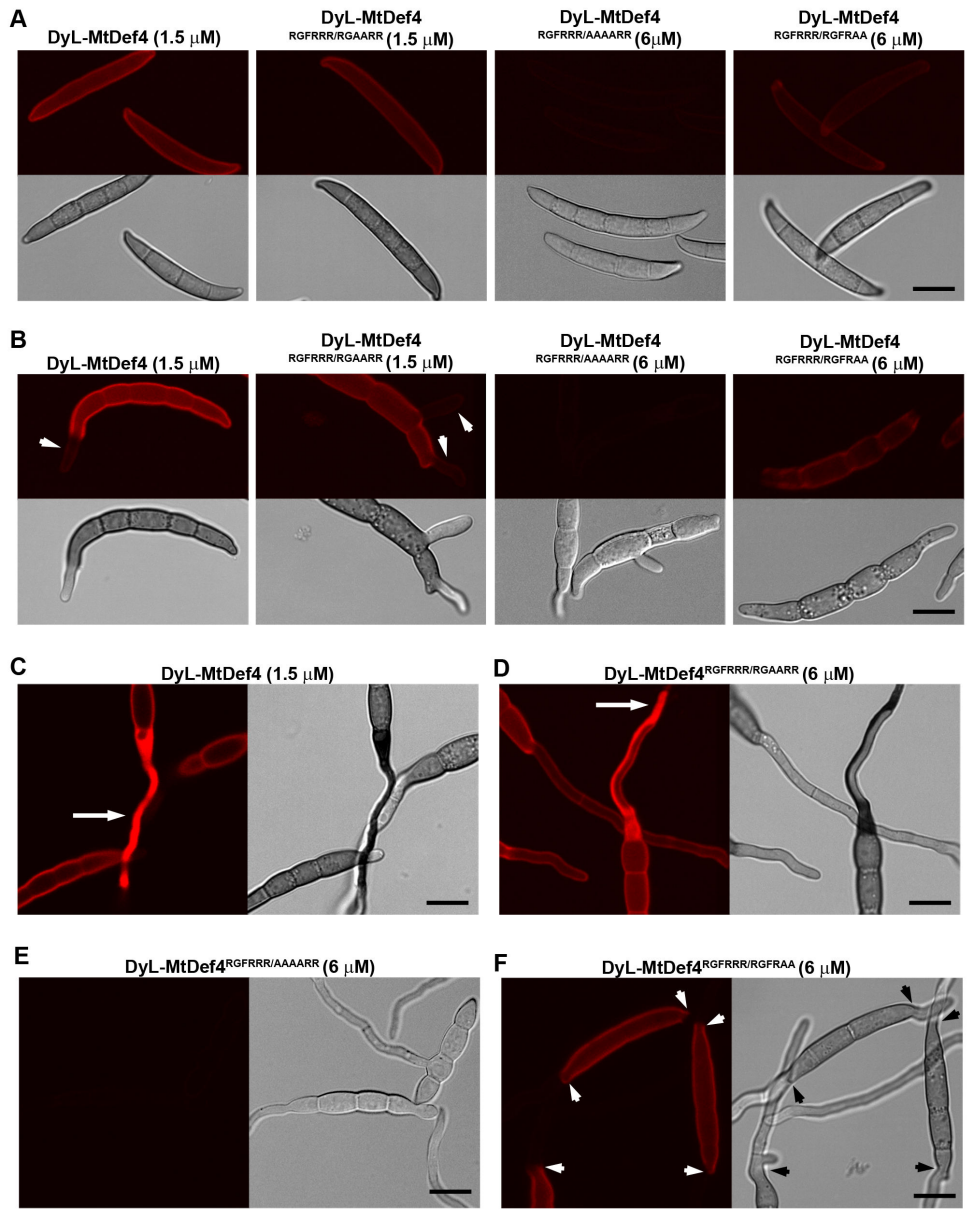
DyLight 550-labeled MtDef4 and MtDef4^RGFRRR/RGAARR^ but not MtDef4^RGFRRR/AAAARR^ and MtDef4^RGFRRR/RGFRAA^ enter *F*. ***graminearum* cytoplasm**. *F*. *graminearum* conidia were incubated with indicated concentrations of DyLight 550-labeled proteins and confocal fluorescence images were taken at various time intervals for up to 6 h. A. Within 15 min, DyL-MtDef4, DyL-MtDef4^RGFRRR/RGAARR^ and DyL-MtDef4^RGFRRR/RGFRAA^ bound to the surface of conidia whereas DyL-MtDef4^RGFRRR/AAAARR^ did not. B. At 2 h, DyL-MtDef4 and DyL-MtDef4^RGFRRR/RGAARR^ bound to the surface of germ tubes but DyL-MtDef4^RGFRRR/AAAARR^ and DyL-MtDef4^RGFRRR/RGFRAA^ did not. C. DyL-MtDef4 entered selective hyphae by 4 h. D. By 6 h, DyL-MtDef4^RGFRRR/RGAARR^ entered hyphae but not all hyphae were affected. E. DyL-MtDef4^RGFRRR/AAAARR^ did not enter the hyphae even after 6 h. F. DyL-MtDef4^RGFRRR/RGFRAA^ bound to the surface of conidial cells but did not bind to hyphal surface. Scale Bar = 10 µm.

Three variants of MtDef4 were next tested for their ability to enter fungal cells. MtDef4 and the variants were each labeled with fluorophore DyLight 550 (Pierce Thermo Scientific, Rockford, IL) in order to follow their entry into fungal cells. The DyLight 550 tagged MtDef4 had significantly reduced antifungal activity as compared with the untagged MtDef4 (Figure S4). Nonetheless, assuming DyLight 550-tagged MtDef4 variants will also have similar loss of antifungal activity, experiments were carried out to compare fungal cell entry of DyL-MtDef4 and DyL-MtDef4 variants. Fungal conidia were incubated either with 1.5 µM and 3 µM DyL-MtDef4 or 1.5 µM, 3 µM and 6µM DyL-MtDef4 variants and were examined at several time points by confocal imaging (Figure 10). Within 15 min, DyL-MtDef4 and DyL-MtDef4^RGFRRR/RGAARR^ readily bound to conidia at a concentration as low as 1.5 µM, while DyL-MtDef4^RGFRRR/AAAARR^ did not bind to conidia even at a concentration as high as 6 µM (Figure 10A). The DyL-MtDef4^RGFRRR/RGFRAA^ variant at 6 µM also bound to conidial surface but with significantly lower intensity than DyL-MtDef4 and DyL-MtDef4^RGFRRR/RGAARR^ (Figure 10A). By 2 hr, a considerable increase in the fluorescence was evident on conidial surface treated with DyL-MtDef4, DyL-MtDef4^RGFRRR/RGAARR^ and DyL-MtDef4^RGFRRR/RGFRAA^, whereas no fluorescence was apparent on conidia treated with DyL-MtDef4^RGFRRR/AAAARR^ (Figure 10B). More importantly, DyL-MtDef4 and DyL-MtDef4^RGFRRR/RGAARR^ readily bound to the surface of germ tubes as they started to emerge from the conidial cells (Figure 10B). In contrast, DyL-MtDef4^RGFRRR/RGFRAA^ bound only to the conidial surface and did not bind to the germ tubes (Figure 10B). This phenomenon was clearly evident in treated cells observed at 4 hr (Figure S5). Images taken after 4 hr showed that DyL-MtDef4 and DyL-MtDef4^RGFRRR/RGFRAA^ were taken up by young hyphae (Figure 10C and D; Movies S1 and S2). It is important to note that internalization of these proteins did not occur uniformly in all treated cells (Figure 10C and D; Movies S1 and S2). At 6 hr, no noticeable fluorescence was observed in cells incubated with 6 µM DyL-MtDef4^RGFRRR/AAAARR^ (Figure 10E), whereas in cells treated with DyL-MtDef4^RGFRRR/RGFRAA^, fluorescence was evident only on the surface of the conidia but not the germ tubes (Figure 10F). These data provide strong evidence that both RGFR and RR sequences are important for fungal cell entry of MtDef4. We conclude that RGFRRR loop mediates fungal cell entry and antifungal activity of MtDef4. 

### Synthetic 16-amino acid peptide containing the RGFRRR loop is internalized by fungal cells, but not the peptide containing RGFR to AAAA mutation

**Figure 11:**
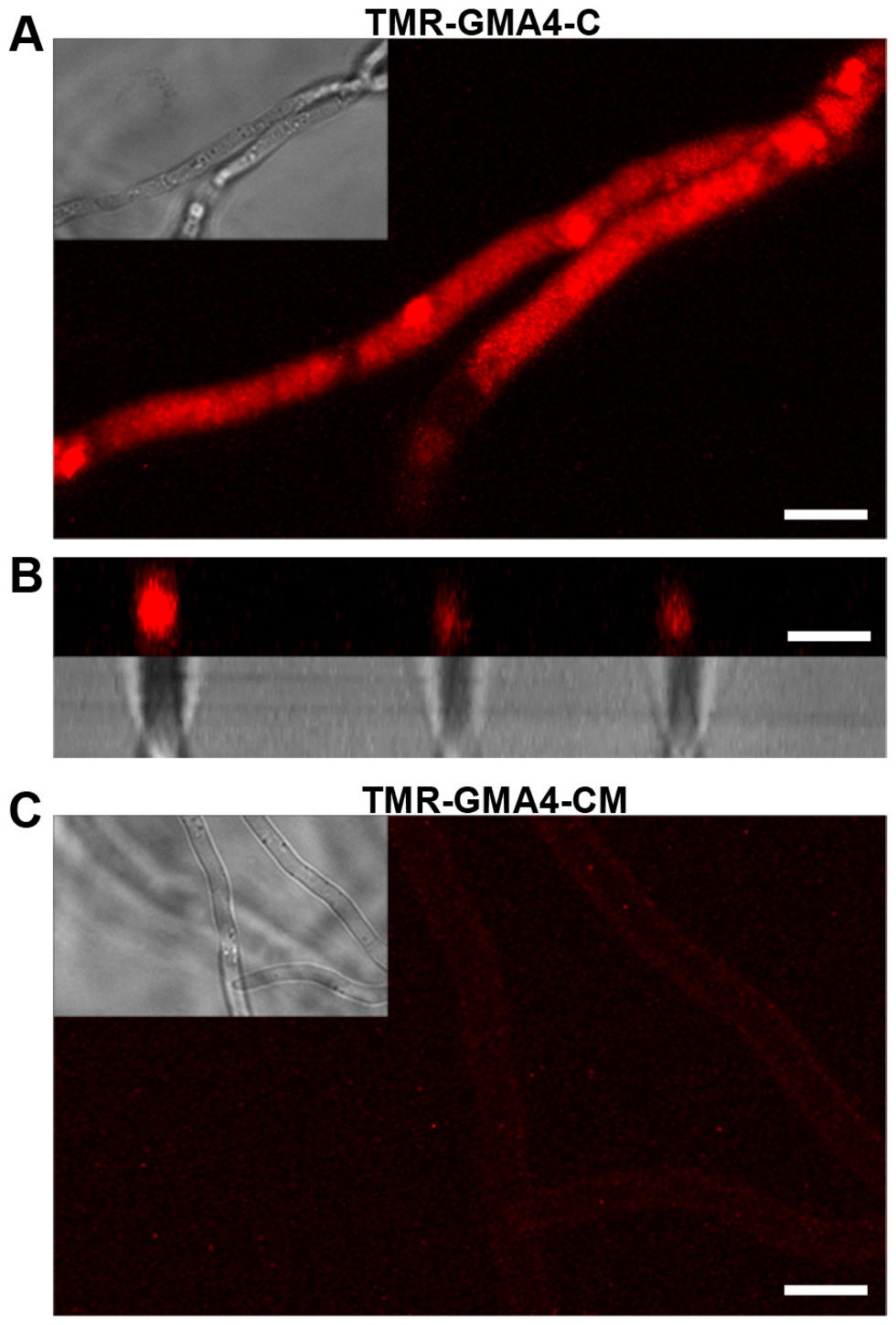
Tetramethyl rhodamine-labeled 16-mer peptide (TMR-GMA4-C) corresponding to the C-terminus of MtDef4 enters *F*. ***graminearum* but a variant peptide (TMR-GMA4-CM) with AAAA replaced for RGFR motif does not**. Fluorescence images of *F*. *graminearum* hyphae incubated with 96 µM TMR-GMA-C or TMR-GMA-CM for up to 4 h. A. TMR-GMA4-C entered the cytoplasm. B. Cross section fluorescent image of selective hyphae from (A) showing that TMR-GMA-C is present in the cytoplasm. C. TMR-GMA4-CM faintly bound to the surface layer but with much lower intensity. Scale Bar = 5 μm.

Previously, chemically synthesized C-terminal 16 residue peptide (GRCRGFRRRCFCTTHC) of MtDef4 containing the RGFRRR loop was found to exhibit antifungal activity [22]. For testing its ability to enter fungal cells, tetramethyl rhodamine (TMR)-labeled peptide, TMR-GMA4-C, containing the carboxy-terminal 16 residues (GRCRGFRRRCFCTTHC) of MtDef4 was chemically synthesized. First, this fluorescently labeled peptide was tested for its ability to inhibit conidial germination and growth. TMR-GMA4-C exhibited considerably less antifungal activity than the unlabeled GMA4-C (data not shown). A variant TMR-GMA4-C peptide, designated TMR-GMA4-CM, in which RGFR was replaced with AAAA (Table 1) was also tested. As expected, TMR-GMA4-CM peptide had significantly lower antifungal activity than TMR-GMA4-C supporting our earlier findings that replacement of RGFR with AAAA in the RGFRRR loop results in significant loss of the antifungal activity of MtDef4 (Figure S6). TMR-GMA4-CM exhibited no antifungal activity even at a high concentration of 96 µM whereas TMR-GMA4-C exhibited significant growth inhibition at 24 µM and completely inhibited conidial germination at 48 µM (Figure S6). These two peptides were therefore tested for their competence to enter fungal cells at 24 µM and above. Confocal microscopic analysis revealed that TMR-GMA4-C when used at 96 µM readily bound to the surface of fungal hyphae within 15 min after treatment (data not shown), whereas no binding was evident at this time point when 48 µM or lower concentrations of the peptide were used. In contrast, TMR-GMA4-CM, even at concentrations as high as 96 µM, failed to bind (data not shown). During the first 2-4 hr of treatment, TMR-GMA4-C (96 µM) profusely bound to the entire length of cell surface and entered the cytoplasm, although the uptake was not observed in all treated hyphae (Figure 11A and B; Movie S3). Even after 4 hr of treatment, TMR-GMA4-CM showed little binding to the cell surface and provided no indication of entry into fungal cells (Figure 11C). Thus, RGFRRR is also required for the fungal cell entry of the 16-residue peptide GMA4-C.

### MtDef4 binds to phosphatidic acid (PA) *in vitro* with high affinity

**Figure 12:**
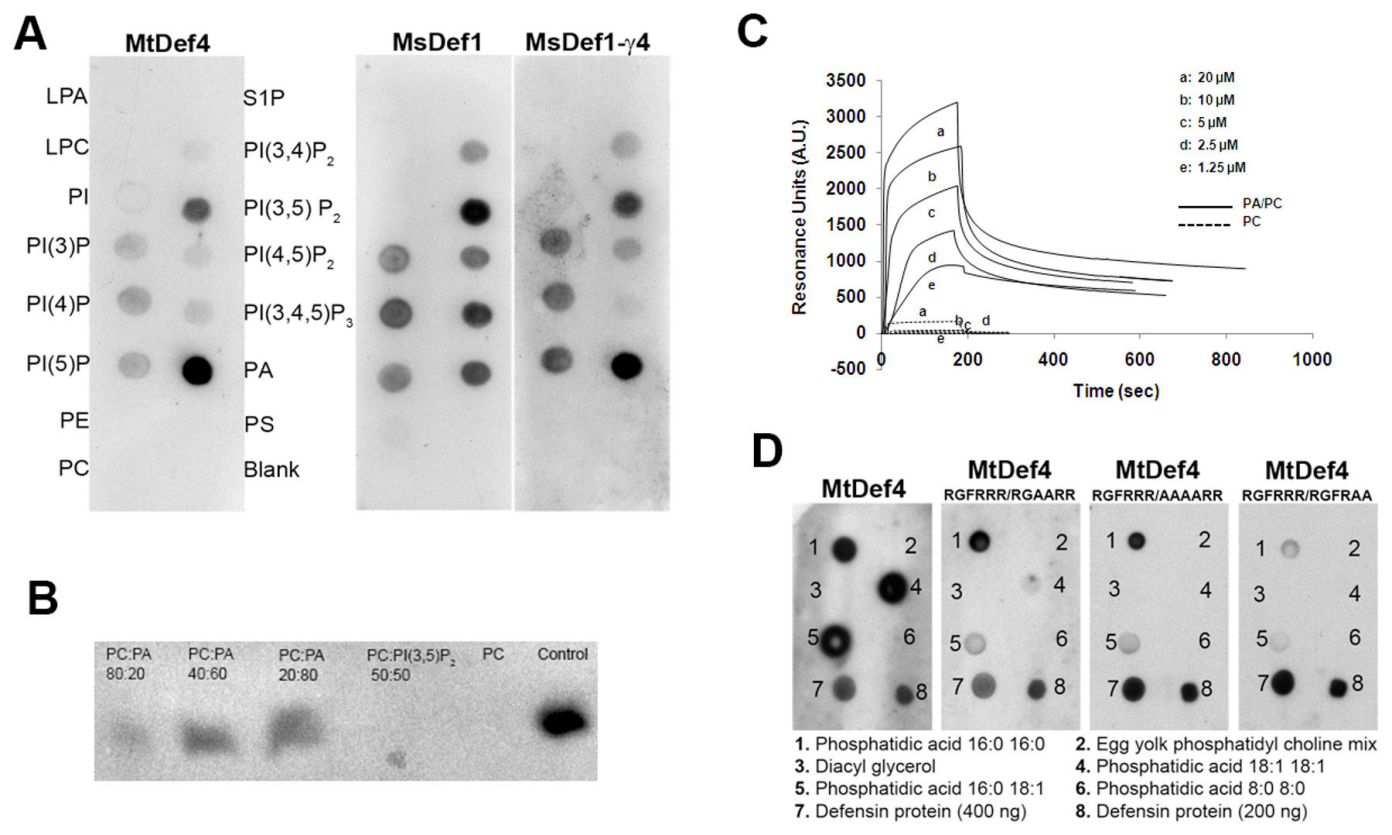
The positively charged RGFRRR loop of MtDef4 plays a vital role in binding to phosphatidic acid. A. Lipid overlay assays of MtDef4, MsDef1 and MsDef1-γ4. P-6001 PIP strips from Echelon Biosciences (Salt lake City, UT) were incubated with desired protein for 1 h at room temperature (see methods for details). After thorough washing, the bound proteins were detected using appropriate rabbit polyclonal-HP antibodies and Supersignal West Pico Chemiluminescent Substrate (Thermo Scientific). LPA = Lysophosphatidic acid; LPC = Lysophosphocholine ; PI = Phosphatidylinositol; PI(3)P = Phosphatidylinositol (3) phosphate ; PI(4)P = Phosphatidylinositol (4) phosphate;  PI(5)P = Phosphatidylinositol (5) phosphate; PE = Phosphatidylethanolamine ; PC = Phosphatidylcholine; S1P = Sphingosine 1-Phosphate; PI(3,4)P2 = Phosphatidylinositol (3,4) bisphosphate; PI(3,5)P2 = Phosphatidylinositol (3,5) bisphosphate; PI(4,5)P2 = Phosphatidylinositol (4,5) bisphosphate; P(3,4,5)P3 =Phosphatidylinositol (3,4,5) trisphosphate; PA = Phosphatidic acid ; PS = Phosphatidylserine and Blank = No lipid. B. MtDef4 binding to liposomes containing PC only or PC plus PA or PI(3,5)P_2_. Purified MtDef4 (2 µg) was incubated with different liposomes for 1 hr at room temperature. The vesicles were pelleted by centrifugation. The protein was visualized by immunoblotting with anti-MtDef4 antibody. 1. PC:PA (80:20), 2. PC:PA (40:60), 3. PC:PA(20:80), 4. PC:PI(3,5)P_2_ (50:50), 5. PC only, 6. MtDef4 (400 ng). C. Surface Plasmon resonance sensograms for the binding of MtDef4 with immobilized PA/PC (3:2) and PC (100%) liposomes. MtDef4 sample dilutions were prepared in PBS buffer and injected at 20 µl/min flow rate. Kinetic parameters were estimated using BIAevaluation software (version 3.1). D. Binding of MtDef4 and its variants to various species of PA. Lipids purchased from Avanti Polar lipids (Alabaster, AL) were spotted (2.5 µg) on Hybond C nitrocellulose membrane. Protein lipid overlay assays were conducted as described above and the bound proteins were detected using anti-MtDef4-HP antibodies. 1. PA 16:0 16:0; 2. Egg yolk phosphatidylcholine mix; 3. Diacylglycerol; 4. PA 18:1 18:1; 5. PA 16:0 18:1; 6. PA 8:0 8:0; 7. MtDef4 or variant protein (400 ng); 8. MtDef4 or variant protein (200 ng).

Several oomycete and fungal pathogens produce effectors containing the RxLR motif that are internalized by the host plant cells. These effectors bind specifically to phosphatidylinositol monophosphates (PIPs) and these interactions have been shown to play important functional roles in the actions of these effectors [38,39]. Since MtDef4 and its homologs in other plants contain the RxLR-like RGFR motif as part of their RGFRRR loop, we tested MtDef4 for its ability to bind several biologically active phospholipids. Lipid-overlay experiments using a commercially available lipid strip P-6001 (Echelon Biosciences, Salt Lake City) revealed strong binding of MtDef4 to the phospholipids phosphatidic acid (PA) and to a lesser extent with PI(3,5)P_2_ (Figure 12A). MsDef1, whose γ-core motif contains the RDDFR loop, (Table 1) was used as a control in this experiment and bound to a different set of phospholipids with more affinity to PI(3,5)P_2_ (Figure 11A). To determine if RGFRRR motif was involved in binding to PA, a variant of MsDef1, MsDef1-γ4, which has a γ-core motif of MtDef4 and exhibits antifungal properties of MtDef4 was also tested (Table 1) [22]. Lipid overlay assay clearly indicated that MsDef1-γ4 shifted its affinity from PI(3,5)P_2_ to PA while retaining similar affinity with other phospholipids (Figure 12A). As expected, MsDef1 did not bind to PA. Strong binding of MtDef4 to PA was further validated with a liposome binding assay. The liposomes were made with a mixture of PA, PC and PI(3,5)P_2_ in varying proportions. No MtDef4 was pelleted with PC-only liposomes or PC-PI(3,5)P_2_ liposomes indicating that MtDef4 binds to PA, but no to PI(3,5)P_2_ (Figure 12B). In separate experiments, MtDef4 binding was observed at a concentration as low as 10% PA (Figure S7). To obtain a quantitative measure of binding of MtDef4 to PA, we carried out surface plasmon resonance (SPR) analysis by immobilizing PA/PC (3:2) and PC only liposomes on an L1 sensor chip (GE Healthcare, NY) using different concentrations of MtDef4 peptide as analytes. When the defensin passed over the sensor chip, a strong binding to PA was observed in a concentration dependent manner (Figure 12C). No binding was observed when PC only liposomes were immobilized on the sensor chip. Collectively, the lipid-overlay, liposome binding and SPR experiments show that MtDef4 binds to PA with high affinity. 

### RGFRRR loop is required for interaction with PA

Since MtDef4 preferentially bound to PA, we tested its binding to several biologically active PAs with different fatty acid chain lengths. Lipid overlay assay revealed that MtDef4 binds to all species of PAs tested (16:0 16:0, 18:1 18:1 and 16:0 18:1) except PA (8:0 8:0) (Figure 9B). PA (8:0 8:0) is water soluble and hence would be dissolved and lost during the washing steps of filter hybridization (see Experimental procedures). PC was used as a negative control and as expected MtDef4 did not bind to it (see Figure 12A). Also, MtDef4 did not bind to diacylglycerol (DAG), a precursor of PA in these experiments (Figure 12B). Three variants of MtDef4 were tested to determine if RGFRRR is required for interaction with PA. These variants had either significantly reduced binding or completely lost the ability to bind to two species of PA (18:1 18:1 and 16:0 18:1). Interaction with PA 16:0 16:0 was retained in MtDef4^RGFRRR/RGAARR^ and MtDef4^RGFRRR/AAAARR^ but completely lost in MtDef4^RGFRRR/RGFRAA^ (Figure 12B). Collectively, these data indicate that the RGFRRR loop containing positively charged arginine residues mediates strong interaction with PA by MtDef4 and that this interaction is lost in MtDef4 variants lacking the ability to enter and kill fungal cells. However, the precise molecular mechanism by which RGFRRR / PA interaction mediates antifungal action of MtDef4 remains to be determined. 

## Discussion

The data presented here provide deeper insight into the structure-activity relationships and mode of action of a potent antifungal defensin MtDef4 from *M. truncatula*. TEM immunogold localization experiments as well as confocal microscopy of fungal cells treated with fluorescently-labeled MtDef4 have revealed that MtDef4 is internalized by *F. graminearum* cells and dispersed in the cytoplasm. MtDef4 also induces the granulation and shrinkage of the cytoplasm as has been reported previously for the plant defensin NaD1 [18]. Thus, MtDef4 is a fungal cell penetrating defensin and most likely inhibits fungal cell growth by interacting with intracellular targets and processes. It will be of significant importance to determine if MtDef4 and two other defensins, NaD1 and Psd1, which have been previously reported to be internalized by fungal cells [17-19], affect the same intracellular targets and processes. Additionally, the SYTOX Green uptake experiments reported here reveal that MtDef4 permeabilizes the plasma membrane of *F. graminearum* within minutes [17-19]. However, in the model fungus *Neurospora crassa*, no uptake of this dye was observed suggesting that MtDef4 does not permeabilize the plasma membrane of this fungus even though it is internalized into the fungal cells and causes cell death at low concentration (IC_50_<0.1 µM) (El Mounadi and Shah, unpublished data). The antifungal activity of MtDef4 against *F. graminearum* might be due to its ability to permeabilize the plasma membrane rapidly and to gain entry into fungal cells secondarily. To what extent does the entry of this defensin into fungal cells play a role in the eventual fungal cell death remains an open question.

The key issue of which sequence motifs are required for the entry of MtDef4 into fungal cells has been addressed in the present study. To this end, the structure of MtDef4 has been determined. Like other defensins whose tertiary structures have been deciphered before [25,40-44], MtDef4 also has a general fold of the CSαβ motif. However, the major differences between these plant defensins lie in a very small β2-β3 loop region of the protein which constitutes the γ-core motif, the hallmark feature of the three-dimensional structure of disulfide-containing antimicrobial peptides from evolutionarily diverse organisms [20]. One distinct feature of the γ-core motif of MtDef4 is that it is highly charged with four arginine residues in the 6-residue loop. We have previously reported that this γ-core motif contains major determinants of the antifungal activity and morphogenicity of this protein [22]. MsDef1-γ4 variant in which the γ-core motif of a related defensin MsDef1 was replaced with that of MtDef4 displayed antifungal properties nearly identical to those of MtDef4. In addition, a chemically synthesized peptide (GMA-4C) containing the C-terminal 16 residues and including the γ-core motif was also capable of inhibiting fungal growth [22]. Recently, high resolution X-ray crystallography, X-ray scattering analysis and analytical ultracentrifugation were used to show that antifungal plant defensin NaD1 known to enter fungal cells forms dimers and that this dimerization enhances its antifungal activity [45]. It will be informative to determine using these approaches if MtDef4 is also capable of forming dimers.

Some of the cell penetrating peptides such as the HIV Tat protein and Drosophila antennapedia are known to be rich in arginine residues [46-48]. We hypothesized that the RGFRRR loop within the γ-core motif of MtDef4, because of its high degree of cationicity, is the translocation signal for entry of MtDef4 into fungal cells. The RGFR sequence within this loop closely resembles the well-studied RxLR motif present in the amino acid sequences of several oomycete effectors translocated into plant cells [34,36,49]. We have recently found MtDef4 homologs in cassava which contain the RxLR motif instead of the RxFR motif found in MtDef4 (Figure S1). Mutagenesis experiments clearly show that the RGFR and carboxy-terminal RR sequence of the RGFRRR loop are required for the entry of MtDef4 into fungal cells. Collectively, the data presented here provide strong evidence that RGFRRR loop is the translocation signal mediating entry of MtDef4 into fungal cells. Bioinformatics analysis of the MtDef4 homologs from other plants indicates that RGFRRR loop is conserved in a large majority of these proteins. However, as shown in Figures 8A and S1, some sequence flexibility exists in this motif. Thus, arginine at position 1 of this loop is replaced with lysine or histidine. Phenylalanine at position 3 is replaced with leucine or valine. Arginine at position 4 is replaced by histidine or threonine and arginine at position 6 is replaced with lysine in some homologs of MtDef4. Whether these amino acid replacements still allow the corresponding defensin to be internalized by fungal cells remains to be determined. In order to fully elucidate the MtDef4 entry route into fungal cells, use of live-cell imaging techniques available in model filamentous fungi such as *Neurospora crassa* is needed [50]. 

Although MtDef4 is internalized by fungal cells, the molecular mechanisms by which it penetrates the fungal cell wall and plasma membrane and exerts its antifungal activity are not known. Like other cell penetrating peptides, MtDef4 is likely taken up by fungal cells either through direct translocation or through endocytosis [51]. It is possible that both mechanisms play a role in the internalization process of MtDef4 depending upon its concentration as well as the state of fungal cell growth and differentiation [52,53]. The antifungal defensin NaD1 has been shown recently to be internalized via a novel mechanism that requires the presence of the fungal cell wall [19]. It has been proposed that NaD1 binds to an as yet unidentified receptor located in the proteinaceous layer of the cell wall. Whether entry of MtDef4 into fungal cells also requires the presence of cell wall needs to be studied. Recently published reports on the mechanisms of the entry of oomycete RxLR effectors have revealed that these effectors bind to phospholipid phosphoinositol-3-phosphate (PI3P) known as an intracellular molecule. Recent studies have now established that a conserved positively charged patch present in the carboxy-terminal domain, and not the RxLR motif, mediates entry of the oomycete effectors into plant cells [39,54]. 

MtDef4 binds to the phospholipid PA with high affinity as demonstrated by the lipid strip overlay, liposome binding and SPR assays. Further, the mutations of RGFR to AAAA and of the C-terminal RR to AA almost completely knockout binding of these variants to PA and their ability to inhibit fungal growth *in vitro*. To our knowledge, this is the first example of an antifungal plant defensin binding to PA. PA is known to play important physiological roles in all eukaryotic cells. It serves as a precursor for the biosynthesis of membrane phospholipids and also acts as a pleiotropic bioactive lipid that functions in membrane dynamics and signaling [55]. Several proteins from plant, mammalian and fungal cells have been reported to interact with PA including protein kinases and phosphatases, transcription factors, cAMP-specific phosphodiesterases and others [56-58]. Although there is no clearly defined consensus amino acid sequence motif to which PA binds, short stretches of sequences containing basic residues and/or tryptophan have been implicated in binding [56,57]. Thus, it is not surprising that RGFRRR motif of MtDef4 interacts with PA. For future studies of this interaction, it will be useful to develop an NMR-based solution assay to map any additional PA binding domain(s) of MtDef4. Our data strongly suggest that the interaction with PA plays an important role in the antifungal action of MtDef4. How this interaction contributes to the antifungal activity of MtDef4 at molecular level remains to be determined. We speculate that PA plays an important role in the internalization of MtDef4 by fungal cells and that once inside MtDef4 interferes with key aspects of PA signaling and/or the biosynthesis of certain membrane phospholipids. It is very likely that all MtDef4 homologs containing the RGFRRR motif and referred to as Class I defensins [59] likely bind to PA as part of their antifungal action. Recently reported Class I defensin NaD2 which shares 81% sequence homology with MtDef4 and also contains the RGFRRR motif [59] is also predicted to bind PA. In the present study, we used a lipid strip 6001 which contains eight phosphoinositides and six biologically important lipids. We cannot rule out the possibility that MtDef4 binds to other phosphoinositides or biologically important lipids not present on this strip (Echelon Biosciences, UT) used. The possibility that MtDef4 could bind to fungal sphingolipids other than glucosylceramide also remains to be tested. 

Antifungal plant defensins, although structurally similar, exhibit significant differences in their primary amino acid sequences including their γ-core motifs. These defensins may therefore harbor different lipid specificity. In this study we have identified, for the first time, surface-localized γ-core motif sequence which mediates entry of MtDef4 into fungal cells. It also revealed the interaction of this motif with an important bioactive phospholipid as potentially a novel mechanism of the antifungal action of this defensin. Although nothing is known regarding what happens inside fungal cells after MtDef4 is internalized, it is possible that the surface-exposed sequence motif interacts with intracellular proteins and nucleic acids. Other plant defensins such as NaD1 [18] and Psd1 [17] must use different sequence motif(s) to gain entry into fungal cells and these motifs might interact with different phospholipids and be evolutionarily conserved depending upon the cell wall or membrane constitution of their target fungi.

## Materials and Methods

### Fungal cultures and growth medium

The fungal strain, *F. graminearum* PH-1, was stored in 20% (v/v) glycerol at -80°C and was cultured on complete medium (CM) [60]. For production of conidia, the PH-1 strain from CM agar plates was inoculated into carboxymethyl cellulose medium [61] and cultured for 2-5 days with shaking at 28 °C. *In vitro* antifungal assays were performed using synthetic fungal medium (SFM) without calcium as described previously [37,62].

### *In vitro* antifungal activity determination

To monitor the early visible phenotypic effects of defensins on conidial germination and growth of fungal hyphae (at 14-16 h after treatment with defensins), bright-field images were taken using the transmitted light channel in a Zeiss LSM 510 META confocal microscope. The quantitative fungal growth inhibition by defensins and synthetic peptides was estimated by measuring the absorbance at 595 nm using Spectramax M2 spectrophotometer (Molecular Devices, Sunnyvale, CA) [62].

### Expression and purification of MtDef4 and its variants

Defensin and its variants were expressed in *Pichia pastoris* as described previously (Spelbrink et al. 2004) and purified using ÄKTA FPLC (GE Healthcare Biosciences, Pittsburgh, PA). FPLC fractions were concentrated and either dialyzed or desalted and further purified by reverse-phase HPLC (Beckman Coulter, Brea, CA) using a C18 column (Deltapak Wat 011793, 150 X 3.9 mm, 5 µM, 300 A) to obtain >95% purity. MtDef4^RGFRRR/AAAARR^, MtDef4^RGFRRR/RGAARR^ and MtDef4^RGFRRR/RGFRAA^ variants were generated using QuickChange II XL site-directed mutagenesis kit using the manufacturer’s protocol (Agilent technologies, Santa Clara, CA). Fluorescent peptides derived from defensins (Table 1) were synthesized at Genemed Synthesis, Inc. (San Antonio, TX). Fluorescently labeled MtDef4 and its variants were generated by conjugation with DyLight 550 dye (Pierce, Rockford, IL) using the manufacturer’s instructions. All peptides were purified to >95% homogeneity by reverse phase HPLC and characterized by mass spectroscopy. Concentration of MtDef4 and its variants was determined by BCA assay kit (Pierce, Rockford, IL). Peptide concentration was determined by quantitative amino acid analysis performed at the Proteomics and Mass Spectrophotometry Facility at the Danforth Center. All defensins and peptides used in this study were dissolved in sterile double-distilled water and stored at -20 °C until further use.

### Preparation of ^15^N-labeled MtDef4

MtDef4, expressed in *P. pastoris*, was labeled with ^15^N essentially as described [63]. A 5 ml culture of *P. pastoris* was grown overnight at 30 °C and then increased to 100 ml volume the following morning. For this initial growth phase, FM23 medium was used (0.8% YNB without amino acids and ammonium sulfate, 2mg/l biotin, 1.2% (NH_4_)_2_SO_4_, 0.3% K_2_HPO_4_, 0.28% KH_2_PO_4_, pH 5.5) to which 3% glucose was added. After 24 h from the initial inoculation, an additional 2% glucose was added to the medium. Six hours prior to methanol induction (30-36 h), 0.02% ^15^NH_4_Cl was added. At 36 h, the cells were harvested by centrifugation, washed with a 0.2% glycerol solution, and resuspended in FM23 media containing 1.2% ^15^NH_4_Cl. To allow the cells to adapt to the addition of methanol, 0.2% unlabeled methanol was initially supplied and 0.4% was added every 12 h to 1.4% from 73 to 96 h of growth and induction (see 63 for details). The culture was maintained at 30°C under rigorous agitation for the duration of the experiment and the pH was maintained by the addition of sterile KOH when necessary. 

### NMR spectroscopy

Both ^15^N-labeled and natural abundance MtDef4 NMR samples (~1 mM protein, 20 mM Tris, pH 4.4) were prepared with 93% H_2_O/7% D_2_O and 99.8% D_2_O, respectively. The NMR experiments were conducted at 20 °C on Varian 750- and 600-Inova spectrometers equipped with ^1^H{^13^C,^15^N}ѱ triple resonance probes and pulse field gradients. The NMR data were processed with Felix2007 (Felix NMR, Inc., San Diego, CA) and analyzed with Sparky (v3.115) [64]. Proton and amide nitrogen assignments were made analyzing the data from standard Varian Protein-pack two-dimensional ^1^H-^15^N HSQC, ^1^H-^1^H NOESY, and ^1^H-^1^H TOCSY experiments and three-dimensional ^15^N-edited NOESY and TOCSY experiments. Chemical shifts were referenced to DSS (DSS = 0 ppm) using indirect methods [65]. Distance restraints for the structure calculations were obtained from two separate NOESY experiments using a mixing time of 150 ms: (1) a three-dimensional, ^15^N-edited NOESY-HSQC experiment collected on a ^15^N-labelled sample in 93% D_2_O/7% D_2_O and (2) a two-dimensional, ^1^H-^1^H NOESY experiment collected on a natural abundance sample in 99.8% D_2_O. Conducting the latter NOESY experiment in 99.8% D_2_O made it possible to identify cross peaks that would otherwise be lost near the water resonance in a 93% H_2_O/7% D_2_O sample. Slowly exchanging amides were identified by lyophilizing the ^15^N-labeled NMR sample, re-dissolving it in 99.8% D_2_O, and immediately collecting an ^1^H-^15^N HSQC spectrum (~10 min after the D_2_O addition exchange). To confirm that the sulfhydryl groups of the eight cysteine residues in the protein were all in the fully reduced state, a 32 hr, natural abundance, ^1^H-^13^C HSQC spectrum was collected on the unlabeled sample in 99.8% D_2_O.

### Structure calculations

The cysteine ^13^C^b^ and the majority of the backbone and side chain ^1^H and ^15^N chemical shifts for MtDef4 were assigned and deposited in the Biological Magnetic Resonance Data Bank (BMRB) under Accession number 18345. Structure calculations were performed iteratively using CYANA (v 2.1) [66], the chemical shift assignments and the peak-picked data from the 3D ^15^N-edited NOESY and 2D ^1^H-^1^H NOESY experiments as initial inputs. Twenty-two dihedral angle restraints for Phi (Φ) (-90° ± -30° (α-helix) and -165° ± -115° (β-strand)) and Psi (ψ) (-80° ± -20° (α-helix) and 100° ± 180° (β-strand) were introduced into the calculations on the basis of the elements of secondary structure identified in the early structural ensembles and TALOS calculations [67]. Near the end of the iterative process 34 hydrogen bond restraints (1.8-2.0Å and 2.7-3.0Å for the NH–O and N-O distances, respectively) were introduced in the structure calculations on the basis of proximity in early structure calculations and the observation of 17 slowly exchanging amides in the deuterium exchange experiment. At this same stage, 12 disulfide bond restraints (2.0-2.1Å, 3.0-3.1Å, and 3.0-3.1Å for the S^γ^-S^γ^, S^γ^-C^β^, and C^β^-S^γ^ distances, respectively) were introduced into the structure calculations on the basis of the disulfide bond pattern observed in other defensin proteins (aside from C3-C47, there was some ambiguity assigning the disulfide bonded pairs on the basis of proximity in early structure calculations). In the final set of 100 calculated structures, the 20 with the lowest target function were selected and refined with explicit water [68] with CNS (version 1.1) using force constants of 500, 500, and 1000 kcal for the NOE, hydrogen bonds, and dihedral restraints, respectively, and the PARAM19 force field. For the water refinement calculations, the upper boundary of the CYANA distance restraints was left unchanged and the lower boundary set to the Van der Waal limit. This water-refined ensemble of 20 structures was deposited in the Research Collaboratory for Structural Bioinformatics (RCSB) under PDB code 2LR3. Structural quality was assessed using the Protein Structure Validation Suite (PSVS, v1.3) [69] and are included in the structure statistics summary provided in Table 2.

**Table 2:** Summary of the structural statistics for MtDef4.

*Restraints for Structure Calculations*	
Total NOEs	389
Intraresidue NOEs	120
Sequential (i, i + 1) NOEs	125
Medium-range (i, i + j; 1 < j ≤4) NOEs	57
Long-range (i, i + j; j > 4) NOEs	87
Phi (Φ) angle restraints	22
Psi (Ψ) angle restraints	22
Hydrogen bond restraints	34
Sulfur-sulfur restraints	12
**Structure Calculations**	
Number of structures calculated	100
Number of structures used in ensemble	20
**Structures with Restraint Violations**	
Distance Restraint Violations > 0.05Å	0
Dihedral Restraint Violation > 1°	0
**RMSD to Mean (Å)**	
Backbone N-C^a^-C=O Atoms (Ordered residues)^a^	0.62 ± 0.13 Å
Backbone N-C^a^-C=O Atoms (All residues)	0.96 ± 0.20 Å
Heavy Atoms (Ordered residues)^a^	1.22 ± 0.15 Å
Heavy Atoms (All residues)	2.00 ± 0.24 Å
**Ramachandran Plots Summary of the Ordered Residues using Procheck**	
Most favored regions	90.0%
Additionally allowed regions	9.8%
Generously favored regions	0.2%
Disallowed	0.0%
**Global Quality Scores**	**Z-score (Raw)**
Procheck (all)	-1.66 (-0.28)
Procheck (Φ,Ψ)	-0.31 (-0.16)
MolProbity clash score	-0.83 (13.75)

^a^ All statistics are for the 20-structure ensemble deposited in the Protein Data Bank (2LR3) using the ordered residues (T2-G12, A15-E27, G32-C34, C41-H46).

### Circular dichroism

Circular dichroism (CD) spectra of MtDef4 and its variants were recorded at 25 °C with a JASCO 815 spectropolarimeter equipped with a rectangular quartz cell with a path length of 0.1 cm. Spectra were recorded in the 250-185.5 nm wavelength at 50-nm/s scanning speed with a response time of 0.5 s. Data were collected from seven separate scans and averaged. Base lines were conveniently subtracted. Mean residue ellipticity is expressed in units of degree × cm^2^ × dmol^−1^.

### SYTOX Green uptake assay

SYTOX Green (SG) assays were conducted as described [22] (Sagaram et al., 2011). Briefly, *F. graminearum* conidia (50µL of 5×10^4^ /mL) were allowed to germinate overnight at room temperature in 2X SFM in a black polystyrene 96-well plate (Corning Inc., Corning, NY). After 15-16 hours, mixtures of MtDef4 or its variants (at concentrations of 6, 3, 1.5, 0.75 and 0.375 µM) plus SYTOX Green (Invitrogen, Carlsbad, CA) at a final concentration of 0.5 µM were added to the hyphae in 50 µL volumes and assay plates were incubated in dark. The fluorescence was monitored (488 excitation; 540 emission (530 cut-off)) at regular intervals using Spectramax M2 spectrophotometer. Black polystyrene plates were used to prevent cross transfer of fluorescence. Fluorescence emitted by hyphae with just SG (without defensins) was regarded as background and hence these fluorescence units were subtracted from defensins containing samples before data analysis. 

### Immunogold labeling of MtDef4

*F. graminearum* conidia (5×10^4^/mL) were germinated in 200 µL 2 x SFM in 1.5 mL Eppendorf tubes with gentle rotation for 16-18 hr and then treated with defensins for 4 hr. The germlings were then fixed for immunogold labeling by high pressure freezing by first rinsing them (after pelleting) twice with 200 µL sterile distilled water. The water from the tubes was removed, 20 µL of 0.1% low melting agarose was added. Cells were transferred to a specimen carrier, frozen using a BAL-TEC HPM 010 high pressure freezer and stored in liquid nitrogen until further processing. The cells were then freeze substituted in acetone containing 0.1% uranyl acetate for 5 days at -80°C, warmed to -50°C then rinsed in acetone infiltrated (over three days) and embedded in Lowicryl HM20 resin polymerized by UV. Thin sections were mounted on coated Ni grids. For immunogold labeling, samples were blocked for 30 min in TBST buffer (Tris buffered saline with 0.05% Tween 20, pH 8.0, plus 20% fetal bovine serum albumin), incubated for 2 hr in rabbit anti-MtDef4 antibody diluted to 20 µg/mL IgG, rinsed in three changes of TBST buffer thirty min, incubated in goat anti-rabbit-gold (15 nm) (Ted Pella, cat. No. 15727, diluted 1:50 in buffer) for two hours, rinsed 30 min in buffer and then rinsed in water. Selected sections were post-stained for 10 min in uranyl acetate and 3 min in Sato’s lead. 

For electron microscopy, MtDef4-treated cells were mounted in 1% low melting point agarose and placed in specimen planchettes for high pressure freezing in a BAL-TEC Model HPM 010 high pressure freezer. Samples were then freeze substituted in 0.1% uranyl acetate in acetone at -85°C for five days. After gradual warming to -50°C they were rinsed in acetone and infiltrated with Lowicryl HM20 resin and polymerized at -50°C with UV light. Some sections were post-stained with uranyl acetate and lead salts. Digital images were acquired using a LEO 912 AB energy filter TEM operated at 120 kV. In some cases large fields of view were acquired by montaging.

### Confocal microscopy

Laser-scanning confocal microscopy was used to detect internalization of fluorescently labeled MtDef4, its variants and peptides derived from MtDef4. For tetramethyl rhodamine (TMR) labeled peptides, *F. graminearum* conidia (150 µL of 2.5×10^4^ /mL) were germinated in a 10 mm microwell of 35 mm glass bottom microwell dishes (MatTek Corporation, Ashland MA). Wet filter papers were placed in the petri dishes to prevent drying of the conidial suspension. The bottom of the 10 mm microwell has a No. 1.5 cover glass and hence facilitates direct observation of the sample without mechanical disruption due to sample transfer. After overnight germination, the medium (in which conidia were germinated) was gently removed and fresh SFM mixed with the desired concentration of TMR-labeled peptides was added and confocal images were acquired at regular intervals. For DyLight 550 conjugated full-length MtDef4 and its variants, 2.5×10^4^/mL conidia were mixed with required concentration of defensin in 150 µL volume and added to 35 mm glass bottom microwell dishes and immediately mounted on the microscope for imaging. A Zeiss LSM 510 META confocal microscope was used for all confocal imaging with an Argon-ion or Helium-ion laser as the excitation source. DyLight 550 and TMR labeled peptides were excited using the 543 nm line of a HeNe laser, and detected using a 565-615-nm bandpass filter. Confocal images were analyzed using Bitplane Imaris software. Data were imported into Adobe Photoshop 4.0 for preparation of figures.

### Protein-lipid interactions using lipid blot assays

P-6001 PIP Strips (2x6 cm nitrocellulose membranes) spotted with 100 pmol of all eight phosphoinositides and seven other biologically important lipids were purchased from Echelon Biosciences (Salt Lake City, UT). In some experiments, lipids purchased from Avanti Polar Lipids (Alabaster, AL) were spotted onto Hybond C nitrocellulose membrane. Interaction of MsDef1 or MtDef4 with lipids present on the P-6001 strips was tested using the manufacturer’s protocol with few modifications. Briefly, the membrane was blocked with 5 mL of blocking buffer, PBS-T (8 g/L NaCl + 0.2 g/L of KCl + 1.44 g/L of Na_2_HPO_4_ + 0.24 g/L of KH_2_PO_4_ + 2 mL/L of Tween-20, pH 7.5) plus 2% fat free BSA (Sigma, St. Louis, MO) and gently agitated for one h at room temperature (RT). The blocking buffer was discarded and 10 µg (final protein concentration: 2 µg/mL) of MsDef1 or MtDef4 was added in 5 mL of blocking buffer and hybridized for 1 h at RT with gentle agitation. After hybridization, the protein solution was discarded and the membrane washed three times with 5 mL PBS-T with gentle agitation. Five ml of PBS-T containing rabbit anti-MsDef1 or anti-MtDef4 polyclonal antibodies (500 times dilution) conjugated with horseradish peroxidase were then added and the membrane incubated at RT for 1 h. After three washes with 5ml PBS-T, the membrane was developed using the SuperSignal West Pico Chemiluminescent Substrate Kit (Thermo Scientific) following the manufacturer’s protocol.

### Liposome binding assay

Phosphatidylcholine (PC) (, L-α-phosphatidic acid (PA) and phosphatidylethanolamine (PE) were purchased from Avanti Polar Lipids (Alabaster, AL) and phosphatidylinositol 3,5 bisphosphate was purchased from Cayman Chemical (Ann Arbor, MI). Liposome binding assay was performed as described with minor modifications [70]. Briefly, lipids were mixed in varying molar ratios in chloroform with the final concentration of lipids per sample being 2.5 µmol. The lipids were dried under nitrogen and rehydrated for 1 h in HBS buffer (100 mM NaCl, 20 mM HEPES, pH 7.5 and 0.02% sodium azide). Following sonication, liposomes were centrifuged at 20,000 x *g* for 20 min. The liposome pellet was resuspended in 1 mL of binding buffer (25 mM HEPES pH 7.5, 50 mM KCl, 1 mM CaCl_2_ and 1mM MgCl_2_) and 2 µg of purified MtDef4 was added and incubated for 1 h at room temperature. Liposomes were collected by centrifugation at 16,000 x *g* for 20 min and washed three times in the binding buffer. Pellet was resuspended in SDS-PAGE sample buffer and loaded on a SDS-PAGE gel. Following electrophoresis, samples were transferred on a nitrocellulose membrane and immunoblotted using anti-MtDef4 polyclonal antibody conjugated with horseradish peroxidase obtained from the Animal Facility of Washington University (St. Louis, MO). Signal was detected using TMB Liquid Substrate System for Membranes (Sigma, St Louis, MO) following the manufacturer’s protocol.

### Surface plasmon resonance analysis

All SPR measurements were carried out on a BIAcore X 100 instrument (GE Healthcare) at room temperature in PBS (pH 7.4). All solutions were freshly prepared and filter sterilized (0.22 µm) before use in the SPR. Liposomes composed of PC (100%) or PC/PA (3:2) were prepared as described above with some modifications. Briefly, 5 µmoles of lipids were dried under nitrogen and rehydrated for 1 h in PBS buffer. Rehydrated lipids were then sonicated in a bath sonicator for 30 min and directly used in the SPR experiment. The surface of an L1 sensor chip (GE Healthcare) was preconditioned by injecting 100 µl of 20 mM CHAPS at flow rate 10 µl/min. The first flow cell of the sensor chip was used as a control surface, whereas the second flow cell was employed as the active surface. The coating of the lipid layers gave a response in the range of 6000-6500. After liposome immobilization, 30 µl of 10 mM NaOH at a flow rate of 30 µl/min was used to wash away any unbound liposome. Hundred µl of fatty acid-free BSA at 0.1mg/ml was then injected at a flow rate of 20 µl/min to block nonspecific binding sites at the sensor chip surface. Peptide solutions of MtDef4 at different concentrations (0-20 µM) were then injected over the lipid surface at a flow rate of 20 µl/min. Association and dissociation times for each injection were 180 and 300 s, respectively. Resonance signals were corrected for nonspecific binding by subtracting the background of the control flow cell. After each analysis, the sensor chip surfaces were regenerated by injecting 40 µl of 2 M KCl at a flow rate of 2 µl/min and equilibrated with the buffer before the next injection. Kinetic parameters were estimated using BIAevaluation software (version 3.0). Curves fitting was done with the 1:1 Langmuir binding model.

## Supporting Information

Figure S1 **Amino acid sequence alignment of MtDef4 homologs from different plants.** MtDef4 homologs were aligned using CLUSTAL W. The highly conserved RGFRRR sequence in each homolog is indicated in bold black font. * = Two cassava defensins with RxLR motif which is found in a number of oomycete effectors.
(TIF)

Figure S2 **Circular dichroism spectroscopy of MtDef4 and its variants.** All MtDef4 variants display similar CD spectra indicating that the mutations in the RGFRRR motif did not alter the protein fold and secondary structure.
(TIF)

Figure S3 **MtDef4^RGFRRR/AAAARR^ and MtDef4^RGFRRR/RGFRAA^ are severely affected in their ability to inhibit *F. graminearum* hyphal growth whereas MtDef4^RGFRRR/RGAARR^ has antifungal activity comparable to that of MtDef4.** Images show the inhibition of *F. graminearum* hyphal growth by MtDef4 or its variants at 36 h after incubation of conidia with defensins.  Scale bar = 50 μm.
(TIF)

Figure S4 **DyLight 550 conjugated MtDef4 (DyL-MtDef4) is less potent than untagged MtDef4.** Images showing the inhibition of *F. graminearum* conidial germination and hyphal growth at different concentrations of MtDef4 or DyL-MtDef4. Images were taken after 16 hours of incubation of conidia with defensins. Scale bar = 50 μm.
(TIF)

Figure S5 **DyL-MtDef4 and DyL-MtDef4^RGFRRR/RGAARR^ bound efficiently to *F. graminearum* hyphae by 4 h, whereas DyL-MtDef4^RGFRRR/RGFRAA^ only bound to the conidial cells but not to hyphae.** DyL-MtDef4^RGFRRR/AAAARR^ did not bind to either conidial cells or hyphae. Conidia were incubated with indicated concentrations of DyLight 550-labeled proteins and confocal fluorescence images were taken at 4 h.
(TIF)

Figure S6 **TMR-GMA4-CM, a variant 16-mer peptide that has RGFR to AAAA replacement has significantly lower antifungal activity.** Images show the inhibition of *F. graminearum* conidial germination and hyphal growth at different concentrations of TMR-GMA4-C and TMR-GMA4-CM. Images were taken after 16 h of incubation of PH-1 conidia with peptides. Scale bar = 50 μm.
(TIF)

Figure S7 **MtDef4 binding to liposomes containing PC/PE only or PC/PE plus PA.** Purified MtDef4 (2 µg) was incubated with different liposomes for 1 hr at room temperature. The vesicles were pelleted by centrifugation. The protein was visualized by immunoblotting with anti-MtDef4 antibody.
(TIF)

Movie S1 **DyL-MtDef4 (6 µM) is internalized by *F. graminearum* cells.** Time point: 6 h of incubation.
(MOV)

Movie S2 **DyL-MtDef4^RGFRRR/RGAARR^ (6 µM) is internalized by *F. graminearum* cells.** Time point: 6 h of incubation.
(MOV)

Movie S3 **TMR-GMA4-C, the TMR labeled 16-mer peptide corresponding to the C-terminus of MtDef4, enters *F. graminearum* cells.** Time point: 4 h of incubation.
(MOV)
