# Supplementary figures and images for: Structural and Functional Studies of a Phosphatidic Acid-Binding Antifungal Plant Defensin MtDef4: Identification of an RGFRRR Motif Governing Fungal Cell Entry
Source: PLoS One. 2013 Dec 4;8(12):e82485. doi: 10.1371/journal.pone.0082485 (PMC3853197; doi:10.1371/journal.pone.0082485)

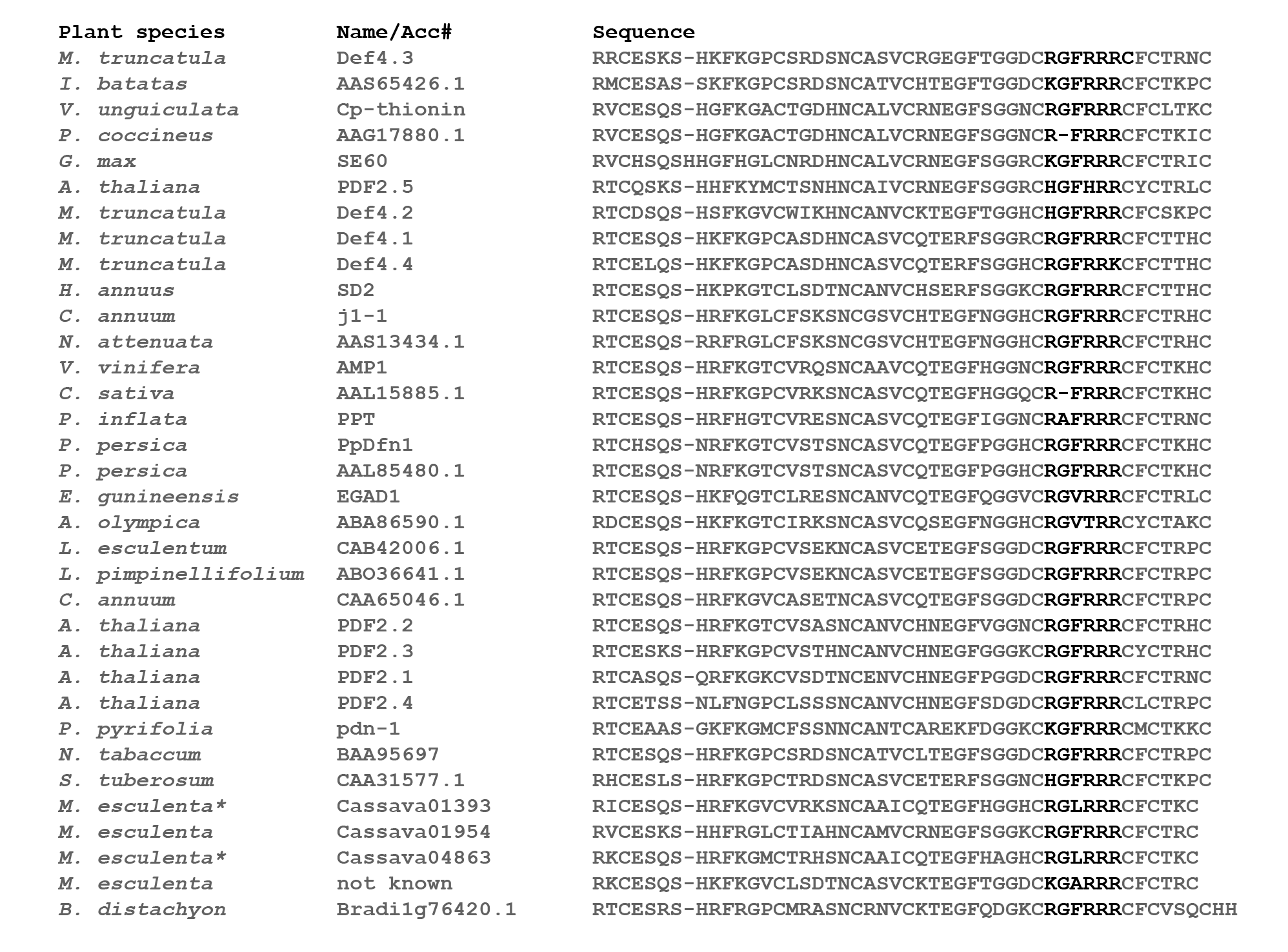

Supplement: Figure S1 — Amino acid sequence alignment of MtDef4 homologs from different plants. MtDef4 homologs were aligned using CLUSTAL W. The highly conserved RGFRRR sequence in each homolog is indicated in bold black font. * = Two cassava defensins with RxLR motif which is found in a number of oomycete effectors. (TIF) [file pone.0082485.s001.tif]

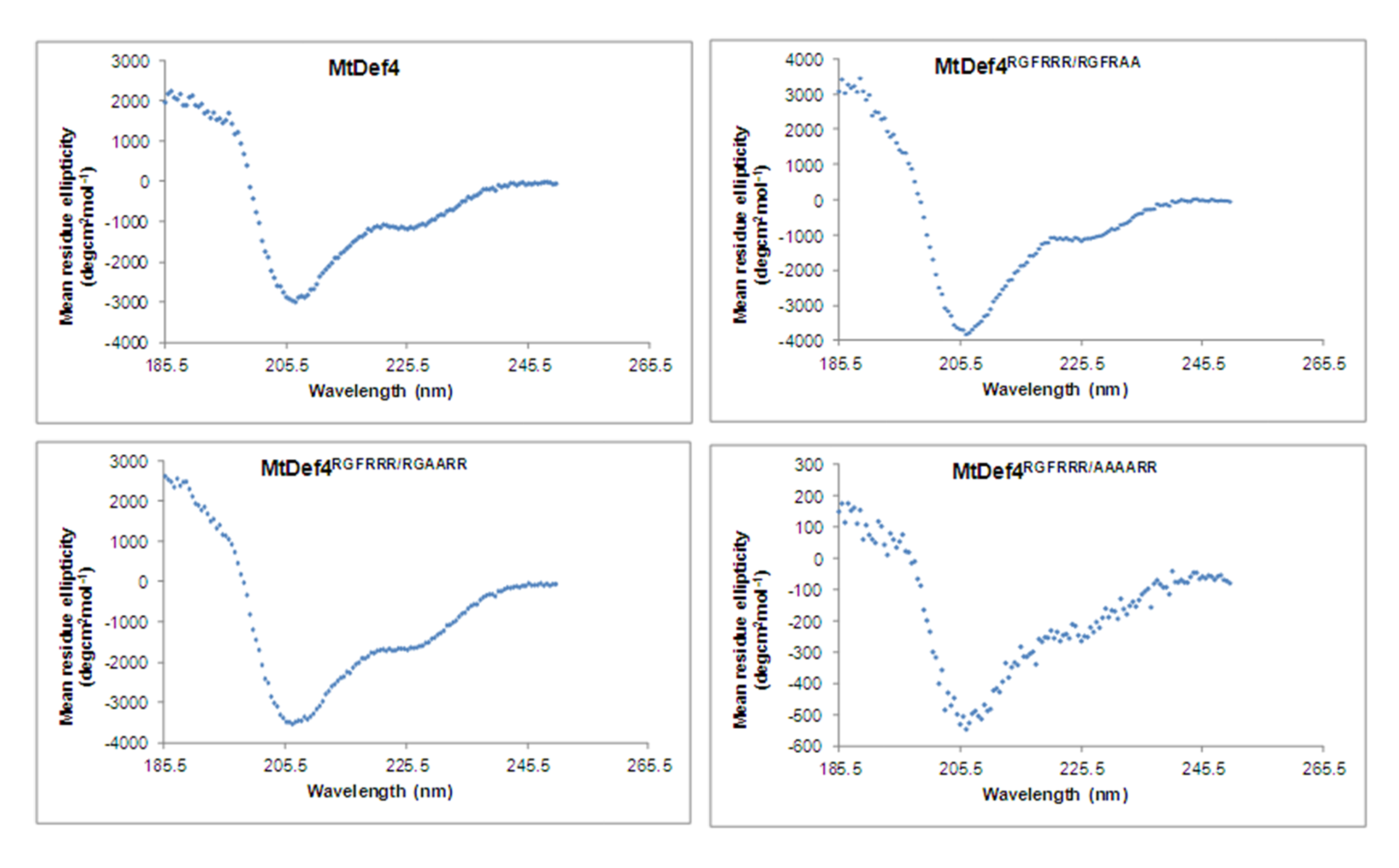

Supplement: Figure S2 — Circular dichroism spectroscopy of MtDef4 and its variants. All MtDef4 variants display similar CD spectra indicating that the mutations in the RGFRRR motif did not alter the protein fold and secondary structure. (TIF) [file pone.0082485.s002.tif]

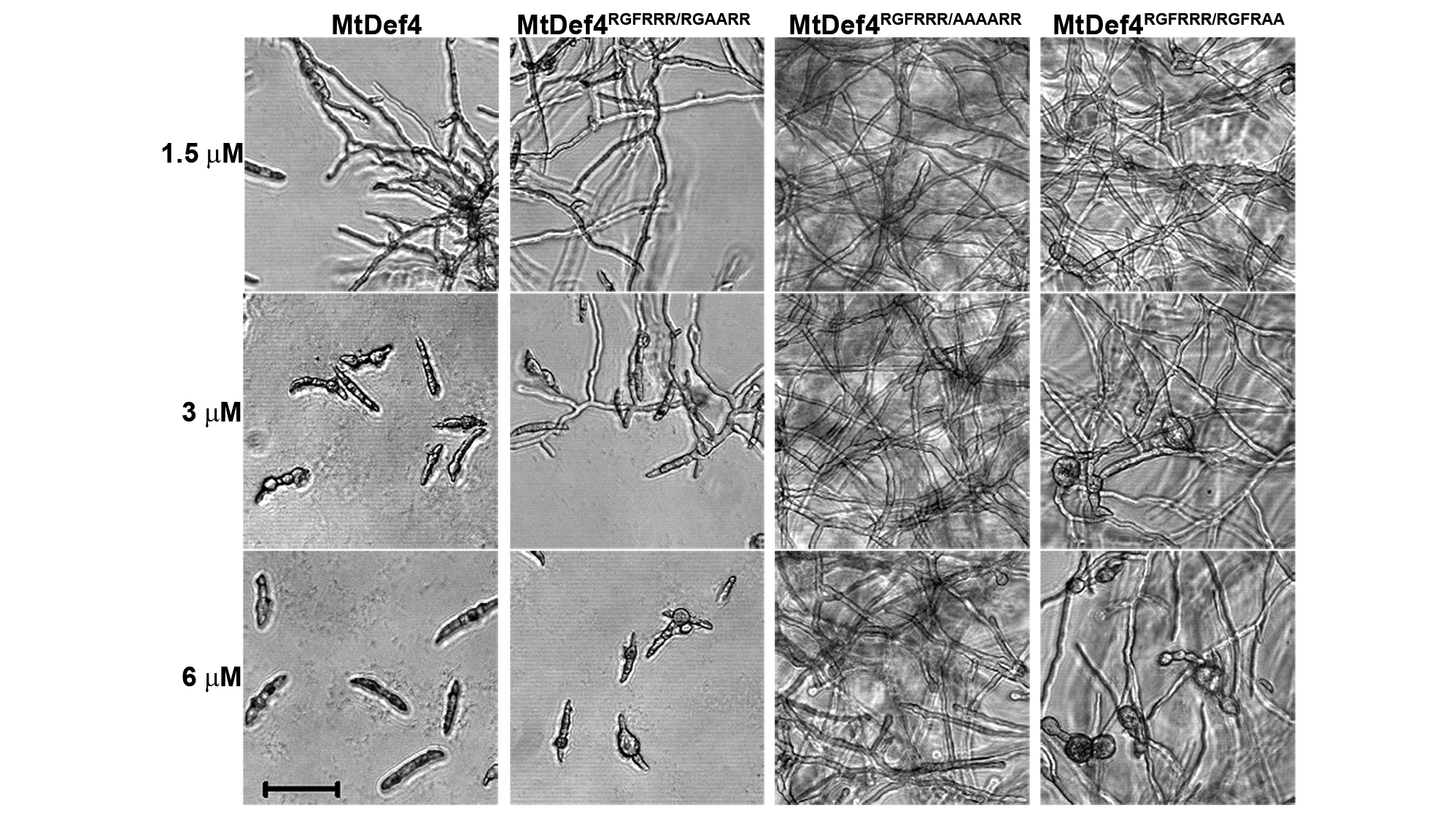

Supplement: Figure S3 — MtDef4RGFRRR/AAAARR and MtDef4RGFRRR/RGFRAA are severely affected in their ability to inhibit F. graminearum hyphal growth whereas MtDef4RGFRRR/RGAARR has antifungal activity comparable to that of MtDef4. Images show the inhibition of F. graminearum hyphal growth by MtDef4 or its variants at 36 h after incubation of conidia with defensins. Scale bar = 50 μm. (TIF) [file pone.0082485.s003.tif]

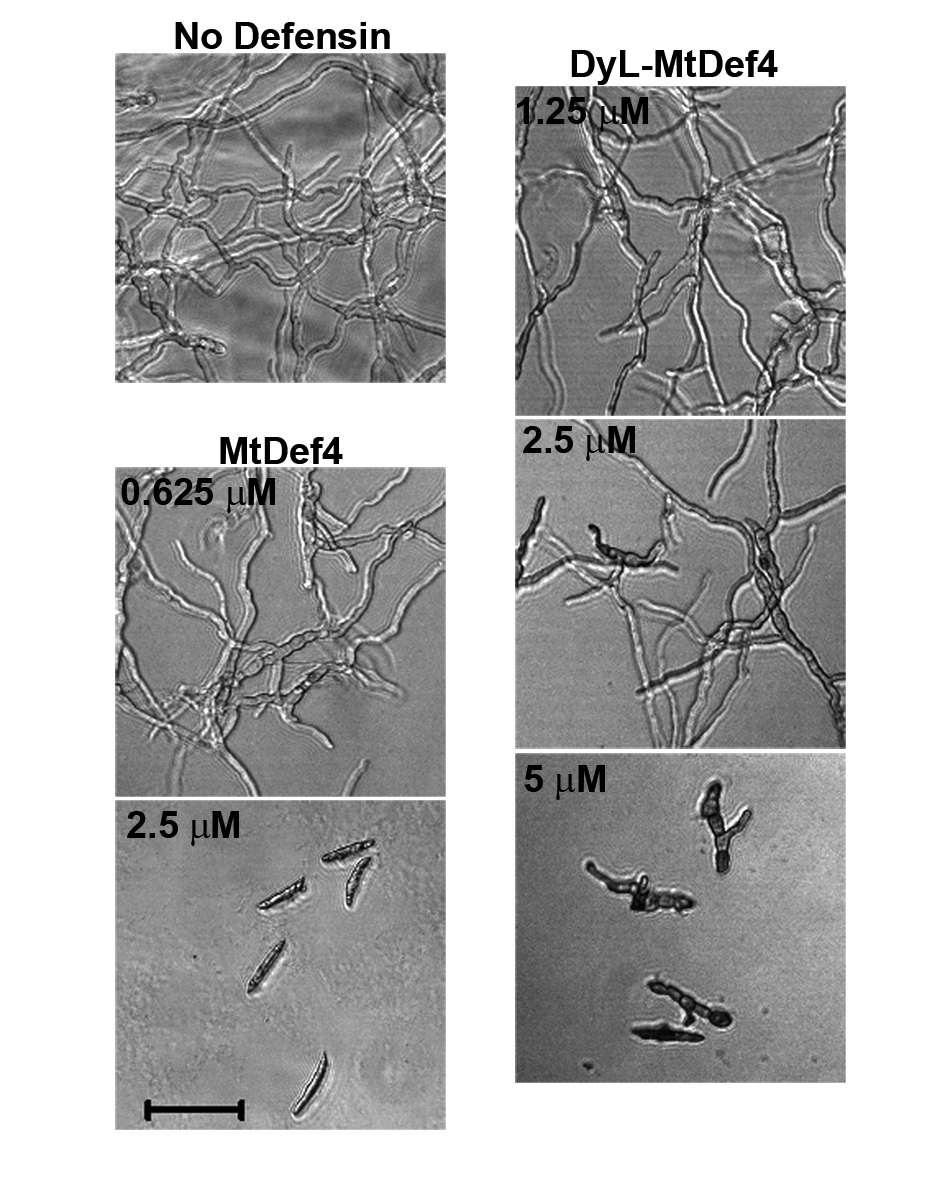

Supplement: Figure S4 — DyLight 550 conjugated MtDef4 (DyL-MtDef4) is less potent than untagged MtDef4. Images showing the inhibition of F. graminearum conidial germination and hyphal growth at different concentrations of MtDef4 or DyL-MtDef4. Images were taken after 16 hours of incubation of conidia with defensins. Scale bar = 50 μm. (TIF) [file pone.0082485.s004.tif]

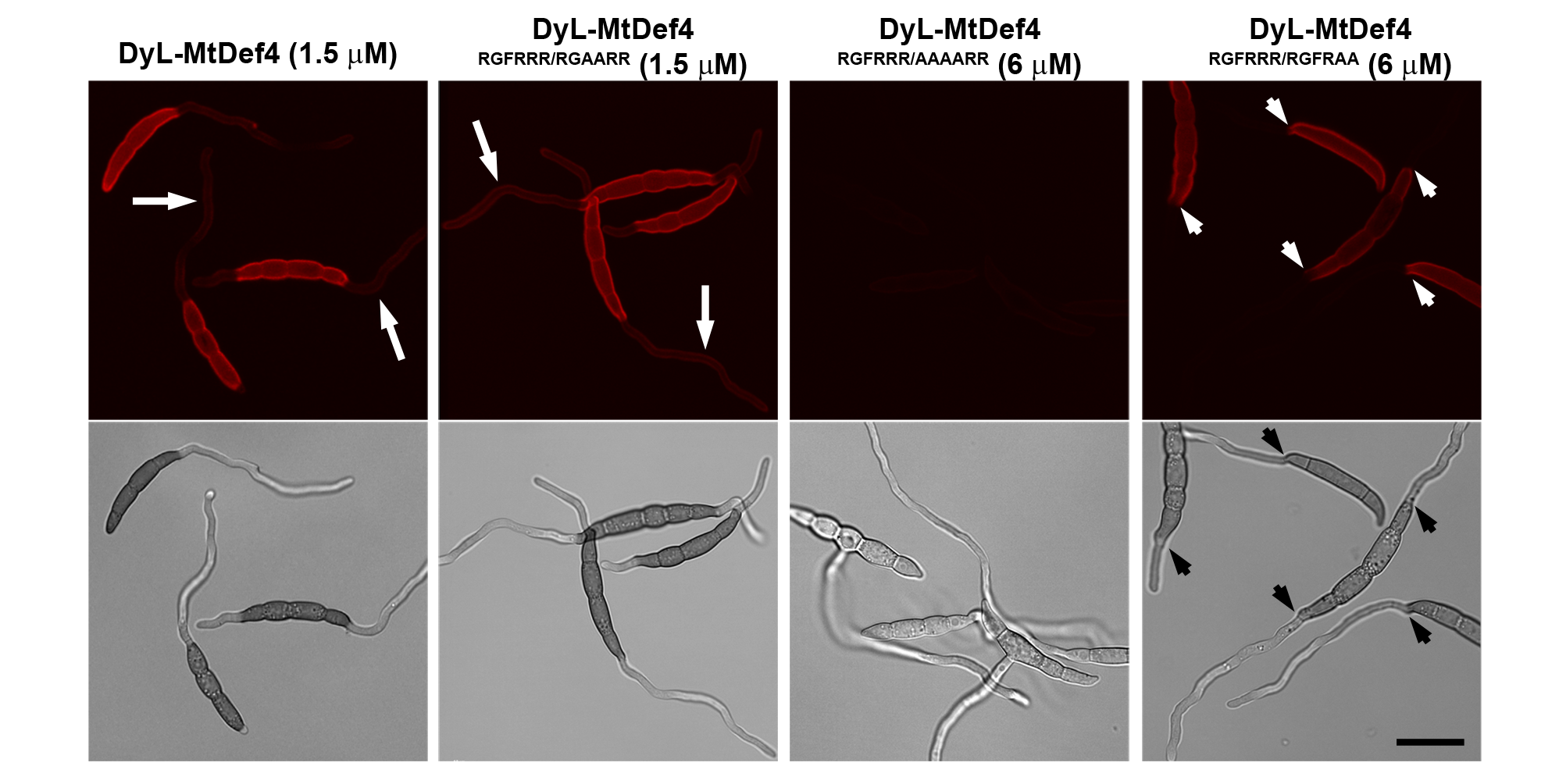

Supplement: Figure S5 — DyL-MtDef4 and DyL-MtDef4RGFRRR/RGAARR bound efficiently to F. graminearum hyphae by 4 h, whereas DyL-MtDef4RGFRRR/RGFRAA only bound to the conidial cells but not to hyphae. DyL-MtDef4RGFRRR/AAAARR did not bind to either conidial cells or hyphae. Conidia were incubated with indicated concentrations of DyLight 550-labeled proteins and confocal fluorescence images were taken at 4 h. (TIF) [file pone.0082485.s005.tif]

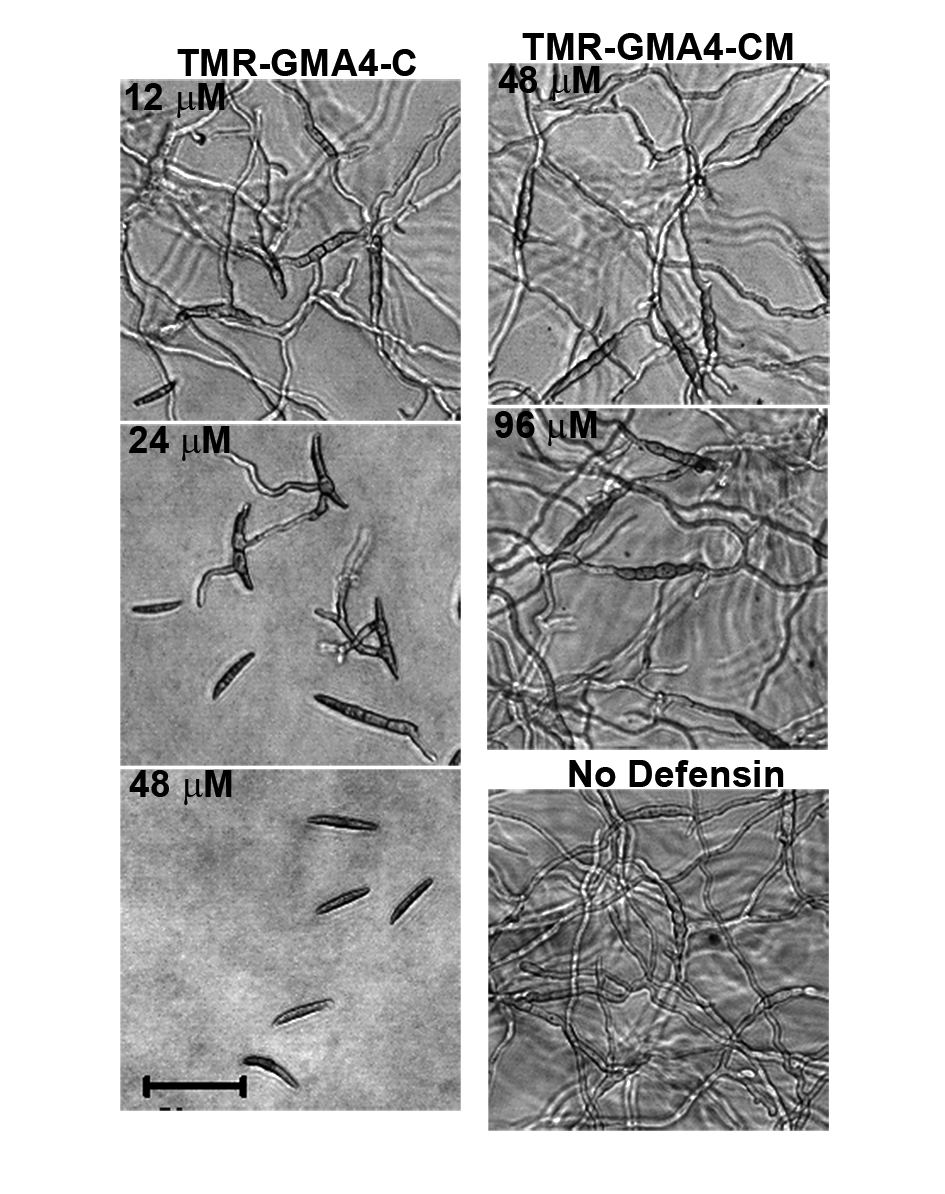

Supplement: Figure S6 — TMR-GMA4-CM, a variant 16-mer peptide that has RGFR to AAAA replacement has significantly lower antifungal activity. Images show the inhibition of F. graminearum conidial germination and hyphal growth at different concentrations of TMR-GMA4-C and TMR-GMA4-CM. Images were taken after 16 h of incubation of PH-1 conidia with peptides. Scale bar = 50 μm. (TIF) [file pone.0082485.s006.tif]

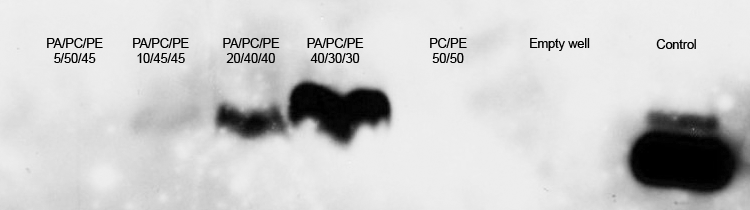

Supplement: Figure S7 — MtDef4 binding to liposomes containing PC/PE only or PC/PE plus PA. Purified MtDef4 (2 µg) was incubated with different liposomes for 1 hr at room temperature. The vesicles were pelleted by centrifugation. The protein was visualized by immunoblotting with anti-MtDef4 antibody. (TIF) [file pone.0082485.s007.tif]
